# EventPointer: an effective identification of alternative splicing events using junction arrays

**DOI:** 10.1186/s12864-016-2816-x

**Published:** 2016-06-17

**Authors:** Juan P. Romero, Ander Muniategui, Fernando J. De Miguel, Ander Aramburu, Luis Montuenga, Ruben Pio, Angel Rubio

**Affiliations:** CEIT, Parque Tecnológico de San Sebastián, Paseo Mikeletegi 48, 20009 San Sebastián, Gipuzkoa Spain; Tecnun, University of Navarra, P° de Manuel Lardizabal 13, 20018 Donostia-San Sebastián, Gipuzkoa Spain; Program in Solid Tumors and Biomarkers, CIMA, University of Navarra, Avda. Pío XII, 55, E-31008 Pamplona, Navarra Spain; Department of Histology and Pathology, University of Navarra, Pamplona, Spain; IdiSNA, Navarra Institute for Health Research, Recinto de Complejo Hospitalario de Navarra, C/Irunlarrea 3, 31008 Pamplona, Navarra Spain; Department of Biochemistry and Genetics, University of Navarra, Pamplona, Spain

**Keywords:** Alternative splicing, Junction microarrays, Protein domains

## Abstract

**Background:**

Alternative splicing (AS) is a major source of variability in the transcriptome of eukaryotes. There is an increasing interest in its role in different pathologies. Before sequencing technology appeared, AS was measured with specific arrays. However, these arrays did not perform well in the detection of AS events and provided very large false discovery rates (FDR). Recently the Human Transcriptome Array 2.0 (HTA 2.0) has been deployed. It includes junction probes. However, the interpretation software provided by its vendor (TAC 3.0) does not fully exploit its potential (does not study jointly the exons and junctions involved in a splicing event) and can only be applied to case–control studies. New statistical algorithms and software must be developed in order to exploit the HTA 2.0 array for event detection.

**Results:**

We have developed EventPointer, an R package (built under the aroma.affymetrix framework) to search and analyze Alternative Splicing events using HTA 2.0 arrays. This software uses a linear model that broadens its application from plain case–control studies to complex experimental designs. Given the CEL files and the design and contrast matrices, the software retrieves a list of all the detected events indicating: 1) the type of event (exon cassette, alternative 3′, etc.), 2) its fold change and its statistical significance, and 3) the potential protein domains affected by the AS events and the statistical significance of the possible enrichment.

Our tests have shown that EventPointer has an extremely low FDR value (only 1 false positive within the tested top-200 events). This software is publicly available and it has been uploaded to GitHub.

**Conclusions:**

This software empowers the HTA 2.0 arrays for AS event detection as an alternative to RNA-seq: simplifying considerably the required analysis, speeding it up and reducing the required computational power.

**Electronic supplementary material:**

The online version of this article (doi:10.1186/s12864-016-2816-x) contains supplementary material, which is available to authorized users.

## Background

Alternative Splicing (AS) has been shown to be a key factor in cellular processes such as development and differentiation as well as in different pathologies, including cancer [1–3]. AS has been studied using Exon arrays and, more recently, using RNA-seq and junction arrays [4].

The first array that made use of junction probes was based on Agilent technology and included approximately 125,000 junction probes, but lacked exon probes [5]. These were included in a later version of the array [6]. In 2010, Oryzon Genomics, in collaboration with our group, introduced an array based on Agilent technology, covering 7,958 genes with a total number of 115,318 exon probes and 105,141 junction probes [7]. This new array made use of a massive number of control probes (as much as 20 % of the array) to ensure the proper normalization of the measurements.

In 2008, Affymetrix presented the Human Junction Array (HJAY). It was their first experimental array with exon and junction probes [8]. This microarray included approximately 6 million probes, comprising ~315,000 exons and ~260,000 junctions. Each exon and junction was interrogated by 8 different probes. The probes of this array were selected using RefSeq, ExonWalk and Ensembl annotations. Two years earlier, ExonHit introduced the Splicearray, also using the Affymetrix technology (although they also provide now a version using Agilent technology). However, the use of Exonhit arrays is not as widespread as the use of standard ones from Agilent or Affymetrix.

In 2011, Affymetrix, together with Stanford University, designed the custom Glue Grant Human Transcriptome Array (GG-H array) [4]. And in 2013, Affymetrix launched the GeneChip® Human Transcriptome Array 2.0 (HTA 2.0), a more up to date catalogue of the HJAY and GG-H arrays. The HTA 2.0 array interrogates a total of 1,048,904 exons or exon clusters with more than 6.3 million probes (approximately 10 probes per exon) and more than 339,000 exon-exon junctions with more than 1.3 million probes (around 4 probes per junction).

In a previous work [9], we developed an algorithm to detect AS cassette events. It was applied to both the HJAY and the Oryzon arrays. HJAY array clearly outperformed the other platform and had a validation rate for top-ranked events of nearly 100 %. The results proved that the Affymetrix platform is a good option to detect AS events. The main problem of this array is that it was unsupported upon their release.

Only the Transcriptome Analysis Console (TAC) 3.0 software offered by Affymetrix and AltAnalyze [10] are the available options to analyze HTA 2.0 and HJAY arrays. FIRMA [11], using CDFs generated by Brainarray [12], can be applied to extract and summarize exon expression but the junction probes would be missing from this analysis pipeline.

The main drawback of the TAC and FIRMA approaches, is that neither of them combines the information provided by the junction probes with the corresponding exon probes in the event under study. For example, in the detection of a cassette event, it is not sufficient to detect the altered expression values of exon probes. In addition to that, the flanking junctions must behave coherently and the skipped junction must have a negative correlation. A similar argument can be made for other AS events. On the other hand, AltAnalyze using the ASPIRE algorithm [13] combines the information of two probesets to get a figure of merit for each event. For example, in a cassette exon, AltAnalyze would provide three figures of merit: one corresponding to the probeset of the exon and the junction that skips it and two more combining the flanking junctions with the junction that skips the exon. Even though, this approach is intrinsically better, it would be still desirable to have a single figure of merit per event. There is an algorithm (MADS+) developed to exploit combined information from exons and junctions [14]. However, its development has been discontinued and it cannot be applied to the HTA 2.0 platform. Furthermore, most of the algorithms developed to detect AS events (including MADS+) are limited to the analysis of case–control studies. Its extension to more complex experimental designs, such as case–control studies with paired samples or time-course studies, is non-trivial.

Here, we present EventPointer, an algorithm to detect AS events using the HTA 2.0 platform. It can be applied to any experiment using appropriately configured design and contrast matrices. EventPointer is based on the limma framework in Bioconductor [15].

## Results

### Implementation

**Fig. 1:**
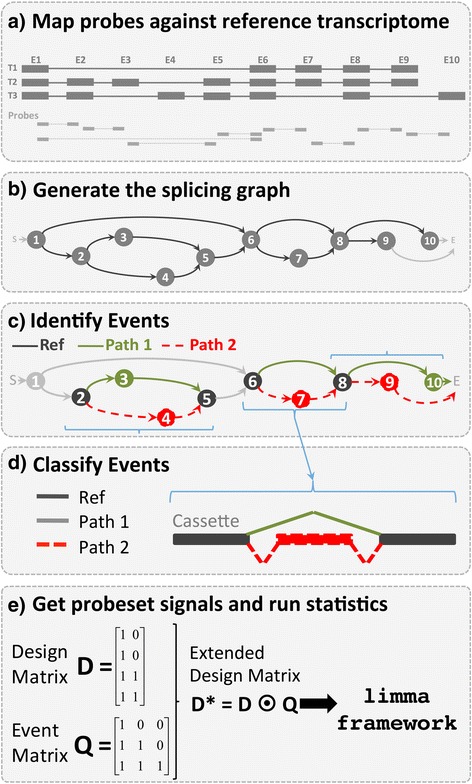
Overview of EventPointer. **a** Using as input a reference transcriptome (Ensembl), the probes of the array are mapped against it, **b** the splicing graph is created. **c** Using the splicing graph, EventPointer detects every possible event by defining common nodes and edges (Reference Path) and two alternative paths (P1 and P2). **d** These events are classified into the canonical splicing categories. **e** Finally, for a specific experiment and using the design matrix of the experiment and an auxiliary event matrix, the statistical significance of each gene is computed using the limma framework. Steps (**a**-**d**) are specific of the array and thus, is only necessary to rerun them if the reference transcriptome is changed. Step E) must be performed for each experiment

**Fig. 2:**
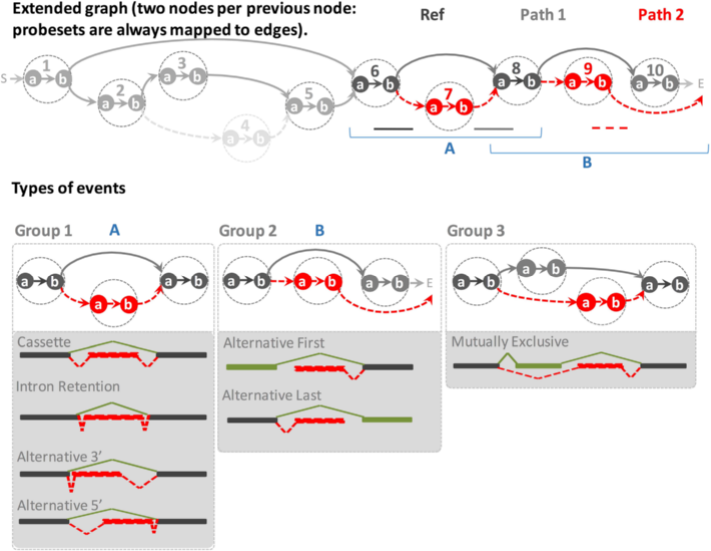
Description of the detection of events from the splicing graph using EventPointer. The SG is extended (every single node is splitted into two to ensure that the probesets are mapped only to edges) and corrected to force. The splicing graph is interrogated to find eventsand detected events are classified into one of the three possible groups (event **a** is a cassette and **b** an alternative last event). Each of these groups are further subdivided into the different event types checking the length of the junctions

Since EventPointer is described in-depth in the Methods section, here we briefly describe its main characteristics. Using Affymetrix junction arrays (the software accepts either HJAY and HTA 2.0), after mapping the probes against the Ensembl transcriptome (Ensembl v. 74) [16], the splicing graph for each gene is generated and EventPointer identifies and classifies the different AS events that can be detected with these arrays. The different classes are alternative 3′, alternative 5′, alternative first exon, alternative last exon, cassette exon, retained intron, mutually exclusive exons and complex events (none of the above). These steps are specific for the arrays and do not need to be repeated for each experiment. The output of this pipeline is a CDF file that groups the probes into probesets that reflect the splicing events. These different steps are depicted in Figs. 1 and 2.

For each experiment, the output of the hybridization (i.e. the CEL files returned by the scanner) must be summarized using any standard pipeline (in our case, RMA). Following probeset summarization (according to the previously prepared CDF file), EventPointer uses the provided design and contrast matrices to compute the statistical significance of AS events. By construction, the AS events can always be validated using standard PCR with at most three primers. We describe here the results of applying the EventPointer algorithm to the HTA 2.0 arrays on an experiment with 27 samples.

The R package EventPointer is available for download at Github. It includes the CDF file needed for aroma.affymetrix pre-processing pipeline and the necessary functions to obtain the statistical results.

**Fig. 3:**
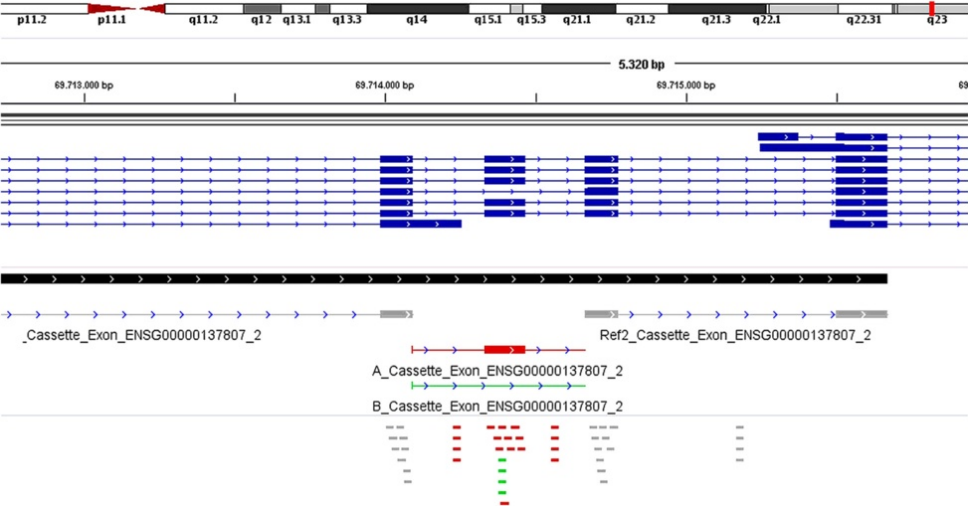
Visualization of EventPointer prediction in IGV. Image generated by EventPointer as displayed in IGV

EventPointer also enables visualization using IGV. EventPointer generates an output file that can be loaded to IGV to display the events (the reference and both paths) as well as the location of the probes for each of the paths. Figure 3 includes a screen capture of an event displayed in IGV.

Within the vignettes that accompany EventPointer we have included some secondary analyses (clustering and functional enrichment analysis of the identified targets) that illustrates the potential of the provided tools.

### Mapping annotations

**Fig. 4:**
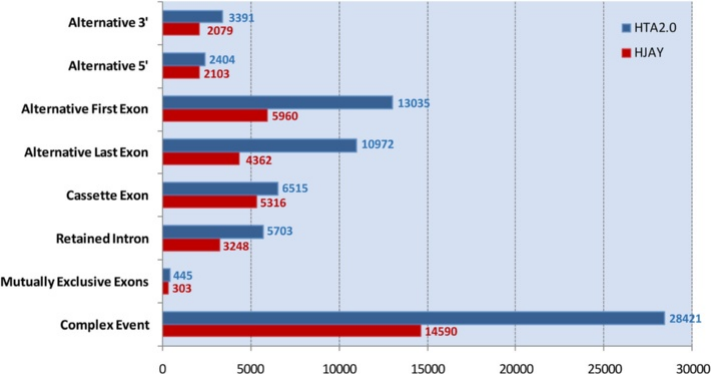
Number of detectable events by EventPointer. Number of AS events detected in each event type for HTA 2.0 and HJAY arrays

The number of events that can be theoretically detected by EventPointer for the HTA 2.0 and HJAY arrays are 70,886 and 37,069, respectively. Figure 4 shows the number of events using the different canonical categories.

As Fig. 4 shows, HTA 2.0 includes more splicing events than HJAY for all event types. The majority of detected event types correspond to complex events (i.e. events that cannot be included in any of the standard categories). As the transcriptome annotation improves, it also becomes more complex. Previously we developed ExonPointer, an algorithm to detect AS cassette exon events [17]. EventPointer extends ExonPointer more than ten-fold, taking the 6515 cassette events in the HTA 2.0 array to more than 70,000 splicing events of any type. In addition to that, EventPointer provides a more rigorous definition of a cassette event and can be run on the HTA 2.0 arrays. We will focus on the results with HTA 2.0 since it interrogates more exons and junctions than HJAY and is a more recent and stable development of Affymetrix.

### Transcriptome data

The performance of the developed algorithms was tested in an experiment where the splicing factor SRSF1 was knocked down using siRNA on the A549 lung adenocarcinoma cell line. This cell line was obtained from the American Type Culture Collection (ATCC). The experiment included three conditions: cells treated only with the vehicle of the transfection (Lipofectamine 2000, Invitrogen), cells treated with scramble siRNA (i.e. a sequence that will not lead to the specific degradation of any cellular mRNA) and cells transfected with a siRNA that targets SRSF1. Each condition had three biological replicates that, in turn, were hybridized three times (9 hybridizations). The efficiency of this inhibition has been stated elsewhere [9].

The splicing factor SRSF1 [18] has a pleiotropic effect: it regulates splicing (enhancing or decreasing certain isoforms), regulates nonsense-mediated mRNA decay, has a role on RNA metabolism (translation), RNA protein binding, has a potential oncogenic role in cancer, regulates the mitosis among other processes [9, 19–21]. We have performed a differential expression analysis of this experiment. We have included the main results in the Additional file 1. The categories of gene ontology enriched are concordant with the aforementioned functions (Additional file 1).

### Determining differential splicing events

An AS event is considered to be differentially spliced if the concentration of the isoforms mapped to either paths of the event (for example, in a cassette event the path that skips and the path that includes the exon) are differentially expressed in opposite directions (i.e. if one of the isoforms is overexpressed, the other must be underexpressed). For this particular experiment, the contrast matrix compares the knock down samples using siRNA with the samples of the cells transfected with scramble siRNA. In the comparison of these two conditions (with siRNA and with scramble siRNA), out of the theoretical 70886 events, 3718 showed a *p*-value < 10^-3^ (approx. 5 % of the events).

Each of the paths in an event (the word path is used here to refer to any of the two configurations of each event; see Figs. 1 and 2, in a cassette event one path skips the exon and the other one includes it) is annotated with the domains (if any) included in the Ensembl database. In some cases, the domain is disrupted in one of the paths compared with the other. We performed a statistical analysis of the enrichment of the domains that may affected by the AS events. The statistics of this analysis are described in the Methods section. In brief, we performed a Wilcoxon test between the isoforms that contain a domain. The results of this analysis are summarized in Table 1.

**Table 1:**
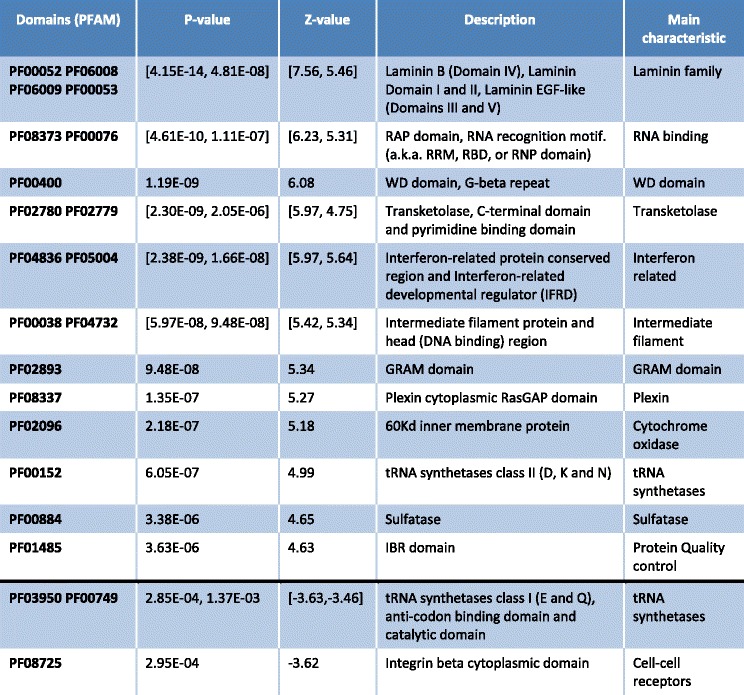
Enrichment of domains in the list of AS events regulated by SRSF1

The domains are sorted by its statistical significance. If several domains share similar properties, are grouped into a single row. The upper part of the table shows overexpressed domains. The second part of the table (divided by a thick line) shows underexpressed domains. The statistical significance was larger for overexpressed than for underexpressed domains

The enrichment analysis illustrates one of the potentials of this analysis. The laminins are proteins of the extracellular matrix. The modification of their domains is known to induce a pro-invasive phenotype [22].

Several of the motifs are related to RNA binding: RNA recognition motif (a.k.a. RRM, RBD, or RNP domain). These results are coherent with the GO enrichment analysis (Additional files 1 and 2) in which several of the enriched categories are related with RNA modification.

To our knowledge, the relation between SRSF1 and the WD40 domain was unknown. The underlying common function of all WD40-repeat proteins is the coordination of multi-protein complex assemblies, where the repeating units serve as a rigid scaffold for protein interactions [23]. In addition to this, the RNA domains are also targets of PRPF8, another splicing factor. IBR, as well as WD40, domains are related to ubiquitin ligase complexes [24].

We have included a few of the domains that were underexpressed after the knock-down of SRSF1. It is important to point out that the statistical significance is much smaller. An intriguing result is that the tRNA synthetases domains are overexpressed and underexpressed depending on their class.

### Validation of EventPointer

We used standard PCR to validate the five top-ranked AS events within each of the eight different types of events (i.e. cassette, mutually-exclusive, complex etc). In total, 40 different events were tested. Each event was validated on two different samples. The events were ranked according to their p-values. In turn, the p-value represents the “differential opposite expression” of each of the isoforms interrogated by an event. The statistical significance of the events (and their ranking) was very different across the different AS types. All the five top-ranked “cassette” and “complex” events were within the overall top-20 ranked events. In contrast, only one out of the five top-ranked “alternative 3′ site” was found within the top-150 ranked events and the top ranked “mutually exclusive exons” was in position 500. These results are summarized in Table 2.

**Table 2:** Top-ranked AS events regulated by SRSF1 and the result of their validation

Ranking	HGNC symbol	Type	Validation
1	MYCBP2	Complex Event	OK
2	KIF23	Cassette Exon	OK
3	AC024560.3	Cassette Exon	OK
4	FBXO22	Cassette Exon	OK
5	SRSF3	Complex Event	~
6	SUPT16H	Alternative Last Exon	OK
7	HMBOX1	Alternative First Exon	OK
8	ACAD11	Complex Event	OK
9	NCOR1	Cassette Exon	OK
10	AUP1	Retained Intron	OK
11	IFT27	Alternative Last Exon	OK
12	GALNT10	Alternative Last Exon	~
13	PARD3	Alternative Last Exon	~
14	PRMT2	Complex Event	OK
15	HORMAD1	Cassette Exon	OK
16	ANAPC7	Alternative Last Exon	OK
17	OGT	Complex Event	OK
23	MSL3	Alternative First Exon	OK
26	NT5C	Alternative 5′	OK
29	ALG2	Alternative First Exon	OK
33	MSL3	Alternative First Exon	OK
39	BAIAP2L1	Alternative First Exon	OK
40	HIST1H2AC	Alternative 5′	~
44	DDX52	Retained Intron	~
71	TMEM214	Retained Intron	~
74	GABPB1	Alternative 5′	OK
77	EIF3B	Retained Intron	~
101	LAMP1	Retained Intron	~
113	LMO7	Alternative 3′	OK
149	SCAMP3	Alternative 5′	X
226	UHRF2	Alternative 5′	X
355	COPS3	Alternative 3′	X
464	SLSC9A8	Alternative 3′	~
473	FLNA	Alternative 3′	X
480	C21orf58	Alternative 3′	OK
500	CALU	Mutually Exclusive	X
688	CCT6P1	Mutually Exclusive	~
844	ST20	Mutually Exclusive	OK
1388	ACO2	Mutually Exclusive	X
1956	KIAA0100	Mutually Exclusive	X

The first column shows the ranking of the event in EventPointer according to the p-value. The last column states if the validation was positive: a (OK) mark is shown if the validation is significant in PCR, a (~) mark is shown if differences in the PCR can be observed but the statistical significance is above 0.05. A (X) mark is shown if the event was not validated either because there was no differential splicing, no alternative splicing or no expression at all

**Fig. 5:**
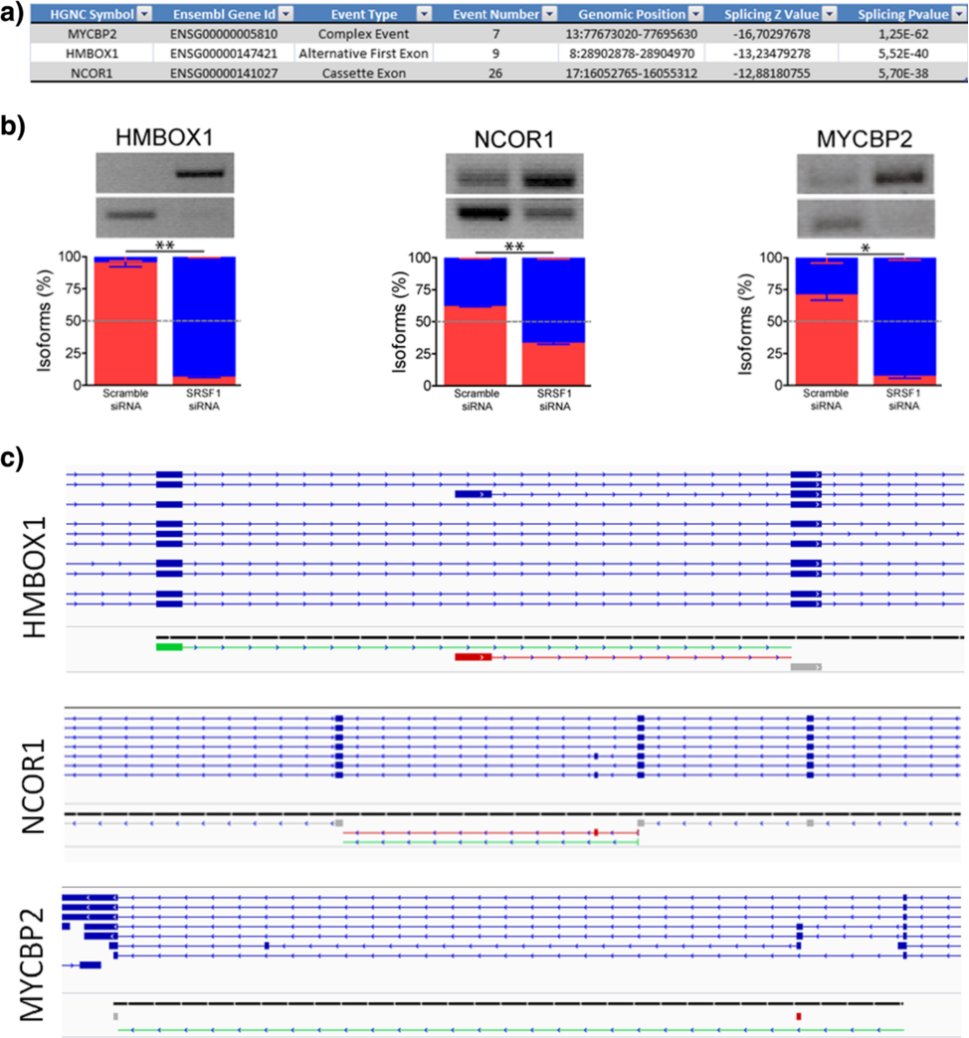
PCR validation of three sample genes predicted by EventPointer. The figure shows the loci in the genome according to Ensembl, the location of the primers, the results of the PCR, and the relative usage of the isoforms determined by a pseudo-quantification of the PCR images

The 17 top ranked events appeared within the top-5 of any of the categories and were validated. In all of them the validation was positive although, in a few cases, the PCR-band analysis (using ImageJ) did not pass statistical significance. In Fig. 5 we show some of the PCR results. Additional file 3 shows the results for all the events. Primer sequences are also included in the additional material (Additional file 4).

### Comparison with other software

Affymetrix Transcriptome Analysis Console (TAC 3.0) and AltAnalyze are the only available software to detect alternative splicing events using HTA 2.0. The main features from each of them are briefly explained below and the comparison with EventPointer is discussed in the latter paragraphs.

#### TAC 3.0

The software from Affymetrix is publicly available for download and provides the user with different tools to go beyond simple differential gene expression analysis. Some of the options are gene pathway networks, miRNA and target gene interactions and alternative splicing events identification. It works only in Windows operating system.

Given the corresponding cel files, the software automatically runs the analysis based on the options provided by the user.

TAC uses Splicing Index (SI) [25] as a measure to detect alternative splicing events. Briefly, the SI of a probeset compares two ratios:

$$ SI = \frac{\left\{\frac{Probeset\ n\  signal\  Cond.\ 1}{Overall\  signal\  of\  the\  gene\  in\  Cond.\ 1\ }\right\}}{\left\{\frac{Probeset\ n\  signal\  Cond.\ 2}{Overall\  signal\  of\  the\  gene\  in\  Cond.\ 2\ }\right\}} $$

If the SI is close to one, then the behavior of the probeset is coherent with the behavior of the gene. If it is much larger or much smaller than 1, then the probeset signal may indicate the presence of alternative splicing.

TAC applies several filters based on expression levels (adjustable by the user) prior to the calculation of the splicing index for any given PSR (probe selection region, i.e. subexon) or junction. It also classifies (some) of the events according to the standard categories (exon cassette, intro retention, etc.). And, for the ones that are classified, TAC includes a “splicing score” (SS). This value is based on “how well the data fits into pre-defined splicing patterns”. Besides, both “PSRs and their related junctions all contribute to an event score” that is bounded between 0 and 1 (from the TAC manual). No additional information is provided on how this score is computed or how the events are classified and can be considered as an experimental method. Only around half of the events are given a SS. In our case, 23/40 (57 %) of the validated events included the SS.

We sorted all the events found by TAC according to the absolute value of the logarithm of the SI (negative values indicate the lack of the exon in the case samples). See Additional file 5 with a list of top TAC predicted events.

#### AltAnalyze

This is an open-source software developed in the Nathan Salomonis lab at Cincinnati Children’s Hospital Medical Center and the University of Cincinnati. This project began in the laboratory of Bruce Conklin at the Gladstone Institutes.. It can be downloaded from their webpage (http://www.altanalyze.org/) and it can be run in different operating systems such as Windows, Mac OS and Ubuntu. As stated by the developers: “requires no advanced knowledge of bioinformatics programs or scripting”.

The software enables analysis of data produced by both conventional and splicing sensitive microarrays (e.g exon and junction arrays) as well as RNA-Seq data and the pipeline enables a complete analysis that includes identification of alternative splicing events and differential expression as well as different functional annotations of the genomic regions identified as alternatively spliced.

For the detection of alternative splicing events, AltaAnalyze uses two different algorithms to measure splicing events: Splicing Index (as in TAC) and analysis of splicing by isoform reprocity (ASPIRE). See Additional file 5 with a list of top AltAnalyze predicted events. AltAnalyze provides the user the option to set different threholds to filter genes and AS events depending on the expression levels.

The ASPIRE algorithm is used when two probesets (A and B) measure the exclusion and inclusion of an exon respectively. It provides a score similar to a fold change, bounded between -1 and 1, where negative values indicate that the expression in the probeset (A or B) in experimental group is higher than the control group. A single splicing event can have (and usually does) several inclusion indexes per event. Each of them correspond to the pairwise comparisons between the probesets that include and exclude the event respectively.

In order to identify an event as statistically significant, AltAnalyze relies on three different values: ratio of inclusion, ratio of exclusion and δI. The first ratios measure the proportion of the inclusion or exclusion of an exon with the mean expression of the whole gene. Both ratios must be in opposite directions to continue the analysis. The δI value measures the difference between both the inclusion and exclusion ratios. As a default value, any event must have a δI above 0.2. A detailed explanation of the algorithm can be found in the AltAnalyze user manual. EventPointer takes into account all the exons and junctions involved in the alternative splicing event to give its statistical significance. The results for EventPointer are normalized using the probeset in the reference path and using the whole gene for AltAnalyze and TAC. On the other side, TAC (using SI) and AltAnalyze (using ASPIRE) provide up to 3 statistical values for each event (skipping junction vs flanking junction 1, skipping junction vs exon and skipping junctions vs flanking junction 2). Both AltAnalyze and TAC are focused on case–control studies. EventPointer can be applied to any experimental design that can be described with a design and a contrast matrix.

**Fig. 6:**
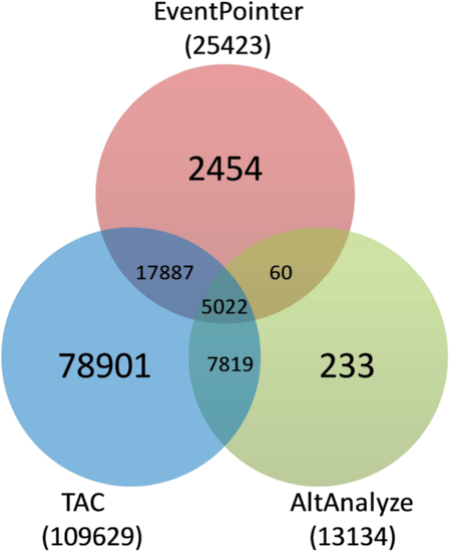
Venn diagram of common events identified by EventPointer, AltAnalyze and TAC The diagram displays the total number of events detected by each algorithm (in parenthesis) and the corresponding values for the intersections

Using the “GRanges” R package [26], we matched the events expressed and detected by the three algorithms (EventPointer, AltAnalyze and TAC). As explained before, an event detected by EventPointer can be matched to more than one element of either TAC or Analyze, as a result we kept the unique events that where matched in order to create the Venn diagram depicted in Fig. 6. This diagram shows the expressed events for all of them. These events do not necessarily show differential usage of the variants.

As already mentioned, TAC provides a different SI for each of the probesets that interrogate an event. We summarized these values taking the most significant SI for each event. Table 3 provides the ranking of the top ten events detected by EventPointer in both AltAnalyze and TAC.

**Table 3:** Ranking of the top-ranked events according to EventPointer and their ranking positions in the other algorithms (EP: EventPointer, AA: AltAnalyze)

Gene	Event type	Genomic position	EP	AA	TAC
MYCBP2	Complex Event	13:77673020-77695630	1	12	383
KIF23	Cassette Exon	15:69713986-69714774	2	11	3
AC024560.3	Cassette Exon	3:197348575-197350253	3	9	113
FBXO22	Cassette Exon	15:76196323-76205608	4	55	1392
SRSF3	Complex Event	6:36566626-36568967	5	2095	280
SUPT16H	Alternative Last Exon	14:21837979-21852105	6	NA	1026
HMBOX1	Alternative First Exon	8:28902878-28904970	7	6	98
ACAD11	Complex Event	3:132297677-132298402	8	396	3371
NCOR1	Cassette Exon	17:16052765-16055312	9	365	5757
AUP1	Retained Intron	2:74754863-74755133	10	NA	10501

### Some comparison examples of top-ranked events

**Fig. 7:**
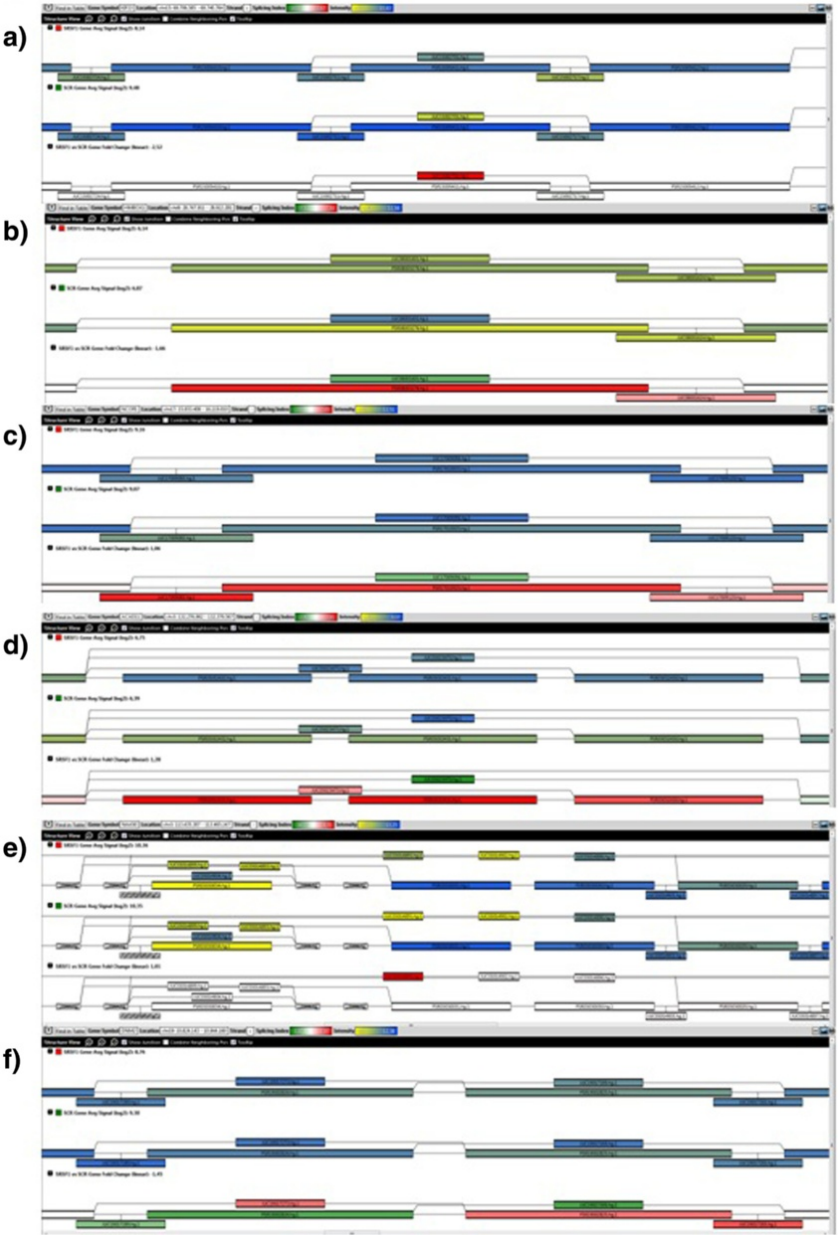
TAC view of six of the compared events. In each of the panels, there are three representations of the splicing graph for each gene. The first one represents the measured expression for each probeset (shown as filled rectangles, yellow: low expression, blue: high expression) in samples treated with SRSF1, the second one represents the expression in the samples treated with scramble siRNA (same color code) and the third one represents the splicing index for each probeset (red: overexpressed, green: underexpressed)

The events that can be clustered into three groups: events with high ranking in the three algorithms, events with high ranking in EventPointer and low ranking in AltAnalyze and TAC and, finally, events low ranked to EventPointer and high ranked in the other two algorithms. The compared events, displayed in TAC, are shown in Fig. 7.

Within the first group, KIF23 was ranked 2nd, 11th and 3rd in EventPointer, AltAnalyze and TAC, respectively. The corresponding statistical values in each algorithm are: 1.00e-58 (EventPointer pvalue), 0.5523 (ASPIRE δI) and 16.67 (TAC SI). The three methods identify this event as high-ranked. Figure 7a shows the TAC window at the particular region for this event.

HMBOX1 is also an event identified as high-ranked by the three algorithms. The corresponding rankings are 7th, 6th and 98th in EventPointer, AltAnalyze and TAC, respectively. Even though the ranking in TAC is not as low as in the other two methods, the PCR validation confirms the alternative splicing event. Figure 7b shows the TAC window for this event.

In the second group, NCOR1 was ranked 9th in EventPointer while AltAnalyze and TAC rank the event in positions 365 and 5757, respectively. This event shows either the highest SI or δI value when compared to the other events found for the same gene. The event was validated by PCR and shows a significant pvalue (5.69e–38) in EventPointer. Figure 7c shows the event in TAC.

ACAD11 is ranked as the 8th event in EventPointer while AltAnalyze and TAC rank the event in positions 396 and 3371, respectively. It shows a similar behavior as NCOR1. Figure 7d displays TAC window for this event.

NAA50 was ranked 10926th,6th and 148th in EventPointer, TAC and AltAnalyze, respectively. The reason is that this event is backed up by only one junction probeset. Since EventPointer finds no coherence with the other paths of the event, the ranking is low (Fig. 7e). This event was validated using PCR. The PCR results are included in the supplementary material (Additional file 6). For this event, the ranking of the event using TAC 3.0 and EventPointer are very different. The reason is the difference on the underlying statistical tests: EventPointer imposes that both isoforms (i.e. both bands in the PCR) must change in opposite directions. In this case, the weak isoform changes its expression (from not being expressed at all to being weakly expressed). However, the most expressed isoform does not change its expression at all and therefore, its statistical significance is low using the EventPointer test. The reason to implement this restriction is that this type of changes (one isoform strongly expressed and a weak change in the other) has a debatable biological implication. Nevertheless, the EventPointer test can also be changed to detect this type of events. If this is done, for this particular case the p.value is smaller than 1e–16 and therefore strongly significant.

DNM2 (2595th, 26935th and 1781th in EventPointer, TAC and AltAnalyze, respectively) is a mutually exclusive exon event. Even though the ranking is not good in EventPointer, it was validated by PCR (see Additional file 7). TAC shows that, although the change in expression between both isoforms is not large, each of the paths has different signs in the corresponding SI (Fig. 7d).

## Discussion

We have presented a complete pipeline to detect AS events using HJAY and HTA 2.0 arrays. The main advantages of this method over the Splicing Index or ASPIRE are that: 1) it can be applied to any experimental design (by providing the corresponding design and contrast matrices) and not only to case–control studies, 2) it exploits the redundancy of all the junction probes involved in an alternative splicing event and 3) it labels all the events according to the different categories. All the suggested events can be validated using standard PCR by construction.

EventPointer is *event*-*focused* instead of *transcript*-*focused* [27, 28]. It estimates the statistical significance of AS events without estimating the concentration of the underlying transcripts. For each event, EventPointer identifies the type of event (cassette, alternative start site, alternative donor, etc.) and provides its statistical significance according to the design and contrast matrices given by the user. EventPointer also generates a graphical output using IGV [29] to make the identification and validation of the events easier.

There are, however, some events that cannot be identified with EventPointer. For example, a couple of isoforms with different transcription start sites in which one of them is included in the other. These types of events are also very difficult to detect using RNAseq or PCR since there is no a specific sequence in the second isoform to design a primer.

In this work, we consider that there is a differential splicing event if the isoforms in the associated paths change their expression in opposite directions. Although this statistical test can miss some AS events, the selected events have a clear change on their expression. These changes, usually, have more biological relevance than other subtler cases in which only one isoform – usually weakly expressed- changes its expression across the conditions.

Once the Affymetrix CEL files are analyzed (i.e. background corrected, normalized, and summarized using the standard procedures), the statistical analysis to detect the AS events is very fast by using the limma package. Using a standard Intel i5 processor, the analysis requires about 10 s. The hardware requirements are modest (a low-end desktop computer with 4GB of RAM is sufficient). The whole enrichment analysis takes only fractions of a second. This is an advantage compared to the requirements on storage, computational power and memory of RNA-seq analysis. In addition to that, the proposed methodology is very reliable: only one false positive was found within the top-200 tested events.

The validation rate is quite high. Our thought is that the reasons for this are on the one hand EventPointer gets a single statistics that combines the information within all the probesets interrogating the event and, on the other hand, uses a proper reference region for each event: most of the methods select a number of probesets (all of them, only the one of constitutive exons, core or quasi-constitutive exons, etc.) that are used for all the events in the gene. However, in our case, this reference is different for each event.

One of the key parts of the analysis of alternative splicing is to provide a biological interpretation of the splicing events, i.e. what is the difference between the isoforms expressed in a condition specific manner.. EventPointer provides the protein family domains that are affected on each of the splicing events. It also performs an enrichment analysis (event-based) to identify which are the domains that are significantly over or underused in the condition under study. Even though in its present form, EventPointer only provides information on the PFAM domains we are actively developing it and we have some alpha versions of the software that provide annotation for other domain databases such us Pirsf, Superfamily, Smart, Prosite, or Interpro. In addition to protein domains, there are other interesting biological data that could be inferred. For example in [30], Ray et al. identify the binding motifs of several hundreds of RNA binding proteins and the potential binding sites in the human genome. Using this information is possible to predict which are the splicing factors that are driving the differential usage of isoforms. Another potential improvement would be to identify miRNA binding sites and check if the splicing pattern causes skipping these binding sites and therefore, the corresponding miRNAs may be no longer regulating the expressed isoforms. This functionality is already offered by AltAnalyze and we expect to include it in the near future. Finally, in its present form, EventPointer works for the HJAY and the HTA2 arrays. We are actively developing the annotation to apply EventPointer to Mouse and Rat junction arrays (MTA-1 and RTA-1).

EventPointer could be extended to RNA-seq by building up the corresponding splicing graph and, in fact, we are currently working on this extension. AltAnalyze can also be applied to RNAseq and has been used for example in [31]. In order to apply EventPointer to RNAseq data, the splicing graph must be constructed based on the sequencing reads and/or the annotation (for microarrays we only use the annotation since the sequences of the probes are fixed). The statistics to perform the analysis must also be changed: instead of using a linear model on the log of the data signal, other methods such as voom [32], or edgeR [33] should be used to take into account the discrete nature of the reads.

There is one potential advantage of EventPointer when extending it to RNAseq experiments. In microarrays, the affinities of the probes are difficult to predict and, usually, they are considered to be unknown. Any algorithm (including EventPointer) get results by implicitly estimating the affinities given the data. The role of affinities in RNAseq is played by the length of the regions that originated the reads (the length of the exon where they came from, for example). EventPointer can be adapted to use the length of these regions to perform the statistics or leave them as unknown and guess them (as it happens with the arrays). This second approach does not require the general assumption of considering uniform coverage of the reads (that is known not to be true).

## Conclusions

We have developed EventPointer software to detect AS events using Affymetrix arrays. It has a high validation rate and shows its effectiveness to detect AS events using Affymetrix splicing-sensitive arrays. In addition to that, its connection with IGV makes it very convenient to validate the results using PCR.

This technique can be used on its own, but also to cross-validate RNA-seq experiments. In addition to that, it provides a statistical analysis of the usage of protein domains and provides a single statistic per event that, to our knowledge, is a novel development for analysis at the transcript/event level.

## Methods

### Sample preparation and PCR validation

Downregulation of SRSF1, expression analyses and microarray hybridization were done as previously described [9]. Samples from two independent experiments were used for validation of splicing events by endpoint PCR. Briefly, RNA was retro-transcribed using PrimeScript RT reagent Kit (Takara). PCR was performed using PCR Master Mix (Promega) using the following program: 94 °C 2 min; 30 cycles at 94 °C 30 s, 57 °C 30 s, 72 °C 30 s; and 72 °C 10 min. Primers used are shown in Additional file 4. The PCR products were loaded in 2 % agarose gels and separated for 40–60 min at 100–120 V. Pictures were taken in a UV transilluminator and the densitometry of the bands was analyzed using Fiji software [34].

### Selection of the events

#### Mapping

The probes included in HTA 2.0 array from Affymetrix are mapped against the human transcriptome (Ensembl 74) using Bowtie 2.0. Multimapping probes, those that map against more than 3 genes, are removed since they are considered to be non-informative. This mapping is used to build the CDF file, i.e. to group the probes according to sets of probes and according to the events (for further information see Additional file 8).

#### Construction of the splicing graph

The Splicing Graph (SG) [35] is a directed graph used to represent the structure of a gene (see Fig. 5a and b). Here, nodes are denoted as subexons (contiguous region of the genome that belong to the same set of transcripts) and in the SG two nodes are connected by an edge if both subexons are contiguous in at least one isoform. Two additional nodes, start and end nodes, are added to the graph and nodes in 5′ (3′) locus of any isoform are connected to them.

The nodes of the SG are duplicated (a and b nodes are used to represent each node) so that both exon and junction probes are represented exclusively by edges in the graph (Fig. 1c). Junction probes connect ‘b’ nodes with ‘a’ nodes and probes mapped against exons connect ‘a’ nodes with ‘b’ nodes.

#### Pruning and recovering of the splice graph

The SGs (one per gene) are very complex and include many edges not supported by any probe on the array. The SG has been pruned to retain only edges with probes mapped to them. In some cases there are edges with no probes mapped to them to ensure the coherence of the graph, i.e. (see Fig. 2 upper panel) any node of the SG is connected with the start and end nodes. For example, although there is no probe mapping against junction E7–E8, the edge from E7 to E8 is not removed to ensure that E7 is connected with the end node. In the contrary, there is no probe mapping against junctions E3–E4 and E4–E5 nor exon E4, and thus they can be removed without affecting the coherence of the SG. For further analysis see Additional file 1.

#### Finding the splicing events

In this work, a Splicing Event is described as a triplet {PR, P1, P2} of subgraphs. These subgraphs are composed of sets of edges and nodes that share the following characteristics: 1) the flow traversing any of the edges of each triplet is identical, and 2) the flow traversing any edge in PR is the sum of the flows traversing P1 and P2. P1 is assigned to be the set of edges with the largest genomic length in the transcriptome and P2 to the shortest one (i.e. in a cassette event, P1 is the path that includes the skipping exon). The detection of the events can be automated using graph theory. The values of the fluxes of all the edges are calculated ensuring that two flows will not be equal by random. Then, the edges with common flow values are grouped in sets. And finally, triplets of sets with one of its flows summing up the other two are grouped together (subgraphs 1 and 2). These last correspond to events. For further information, see Additional file 1.

#### Labeling the type of splicing event

The splicing events can be classified into 7 major categories: cassette exons, alternative 3′donor site, alternative 5′donor site, intron retention, alternative last exon, alternative first exon and mutually exclusive exons. Any event not classified into these is considered to be a complex event. The labelling of an event is carried out according to the structure of the subgraph of the event {PR, P1 and P2} (see Fig. 2 lower panel).

#### Summarizing the events

Each of the events is composed of a triplet of probesets: the probes mapped to path 1, path 2 and the reference. For a specific event, there are isoforms that are not mapped to any of the paths in the event. However, if an isoform hits the event then, by construction, the isoform can be mapped to PR and *only to either* P1 or P2.

Within the analysis, we consider that the signal of a probe on an Affymetrix array is the product of the affinity of the probe and the sum of the concentrations of the isoforms interrogated by the probe. Therefore, if *y*_*ij*_ is the logarithm of signal of the probe i in condition *j* then,

1 $$ {y}_{ij}= \log \left(Aff{y}_i{\tau}_j\right)+{\varepsilon}_{ij}= \log \left(Aff{y}_1\right)+ \log \left({\tau}_j\right)+{\varepsilon}_{ij} $$

where *Affy*_*i*_ is the affinity of the probe, *τ*_*j*_ is the sum of the concentrations of the transcripts interrogated by probe *i*, and *ε*_*ij*_ is an error term.

In EventPointer (as in most other methods that use Affymetrix technology), the values of the probe signals within a probeset are summarized to a single value using the median polish algorithm [36]. We have assumed that the model for a single probe is also valid for the summarized value of the probeset (i.e. the signal in the probeset is proportional to the concentration of the isoforms interrogated by the probeset).

**Fig. 8:**
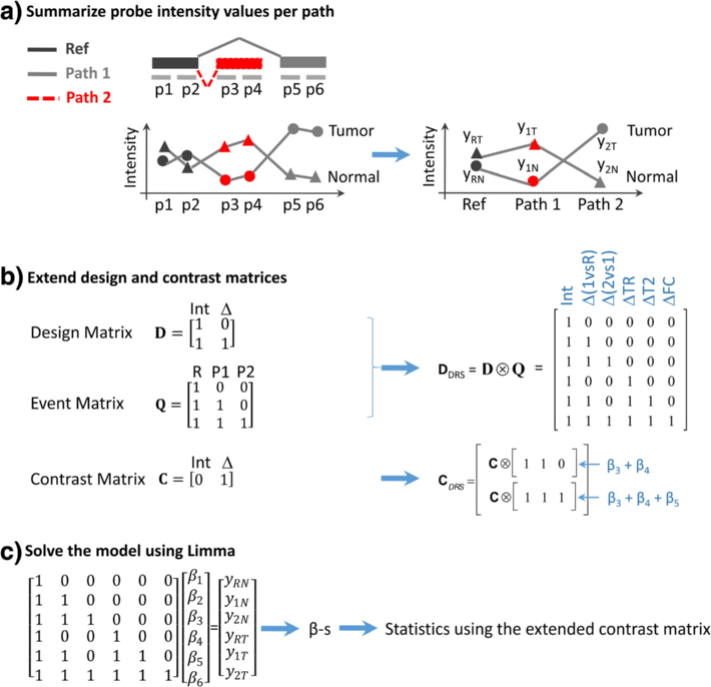
Steps of the statistical Analysis: a toy example, **a** The intensity values are summarized per path and sample, **b** the Design and Contrast matrices defined by the user are extended and (**c**) the linear model is solved to obtain the values of the coefficients and the statistical significance is determined using Limma package

Let us consider a differential splicing event that consists of two alternative stop sites (i.e. transcript end), interrogated by 6 probes. Probes 1 and 2 belong to PR, Probes 3 and 4 to P1, and probes 5 and 6 to P2 (see Fig. 8a). Let us assume that this event is measured in two different conditions, normal (N) and tumor (T). There will be 12 different measurements that, after summarization, are converted to the 6 values *y*_*RN*_, *y*_1*N*_, *y*_2*N*_, *y*_*RT*_, *y*_1*T*_, *y*_2*T*_ that correspond to the summarized signals of PR, P1 and P2 in the normal and tumor samples, respectively (see Fig. 8a). For this example, the signal *y*_*RN*_ -dropping the error terms- (i.e. the probeset in the reference path in the normal sample that comprises probes 1 and 2) is

2 $$ {y}_{RN}= \log \left(Aff{y}_R\left({t}_{1N}+{t}_{1N}\right)\right)= \log \left(Aff{y}_R\right)+ \log \left({t}_{1N}+{t}_{2N}\right) $$

The signal of *y*_2*T*_ (probeset P2 –probes 5 and 6- in the tumor sample), we have,

3 $$ {y}_{2T}= \log \left(Aff{y}_2\right)+ \log \left({t}_{2T}\right) $$

Since the probeset resides in path 2, it only measures the second isoform.

#### Construction of the design matrix for alternative splicing detection

A convenient formalism to describe an experiment is using the contrast and design matrices. This section describes how to build these matrices to detect differential splicing events.

Let **Q** be the following 3x3 auxiliary matrix:

4 $$ \mathbf{Q}=\left[\begin{array}{ccc}\hfill 1\hfill & \hfill 0\hfill & \hfill 0\hfill \\ {}\hfill 1\hfill & \hfill 1\hfill & \hfill 0\hfill \\ {}\hfill 1\hfill & \hfill 1\hfill & \hfill 1\hfill \end{array}\right] $$

Its usefulness will be shown later. Let **D** be the design matrix of the experiment. The proposed design matrix **D**_DRS_ to detect splicing events is:

5 $$ {\mathbf{D}}_{\mathrm{DRS}}=\left[\boldsymbol{D}\otimes \boldsymbol{Q}\right], $$

where ⊗ is the matrix Kronecker product. We have included a simple example to illustrate the concepts. The corresponding design (**D**) and extended design matrices (***D*** ⊗ ***Q***) for the aforementioned example are shown in Fig. 6b. The design matrix includes an intercept term for all the samples and an increment for the tumor sample. If this experiment only studied gene expression, the contrast matrix would simply test if the increment of expression in the tumor samples is different from zero. The Kronecker product is equivalent to replacing each of the entries of matrix **D**, by matrix **Q** multiplied by the corresponding entry in **D**. Given this, the proposed linear model is:

6 $$ \left[\begin{array}{cc}\hfill \begin{array}{ccc}\hfill 1\hfill & \hfill 0\hfill & \hfill 0\hfill \\ {}\hfill 1\hfill & \hfill 1\hfill & \hfill 0\hfill \\ {}\hfill 1\hfill & \hfill 1\hfill & \hfill 1\hfill \end{array}\hfill & \hfill \begin{array}{ccc}\hfill 0\hfill & \hfill 0\hfill & \hfill 0\hfill \\ {}\hfill 0\hfill & \hfill 0\hfill & \hfill 0\hfill \\ {}\hfill 0\hfill & \hfill 0\hfill & \hfill 0\hfill \end{array}\hfill \\ {}\hfill \begin{array}{ccc}\hfill 1\hfill & \hfill 0\hfill & \hfill 0\hfill \\ {}\hfill 1\hfill & \hfill 1\hfill & \hfill 0\hfill \\ {}\hfill 1\hfill & \hfill 1\hfill & \hfill 1\hfill \end{array}\hfill & \hfill \begin{array}{ccc}\hfill 1\hfill & \hfill 0\hfill & \hfill 0\hfill \\ {}\hfill 1\hfill & \hfill 1\hfill & \hfill 0\hfill \\ {}\hfill 1\hfill & \hfill 1\hfill & \hfill 1\hfill \end{array}\hfill \end{array}\right]\left[\begin{array}{c}\hfill \begin{array}{c}\hfill {\beta}_0\hfill \\ {}\hfill {\beta}_1\hfill \\ {}\hfill {\beta}_2\hfill \end{array}\hfill \\ {}\hfill \begin{array}{c}\hfill {\beta}_3\hfill \\ {}\hfill {\beta}_4\hfill \\ {}\hfill {\beta}_5\hfill \end{array}\hfill \end{array}\right]=\left[\begin{array}{c}\hfill \begin{array}{c}\hfill {y}_{RN}\hfill \\ {}\hfill {y}_{1N}\hfill \\ {}\hfill {y}_{2N}\hfill \end{array}\hfill \\ {}\hfill \begin{array}{c}\hfill {y}_{RT}\hfill \\ {}\hfill {y}_{1T}\hfill \\ {}\hfill {y}_{2T}\hfill \end{array}\hfill \end{array}\right] $$

Using some algebra, it is possible to get the value of the coefficients (Table 4).

**Table 4:** Values of the factors for the lineal model and their interpretation

Value of Beta	Interpretation
**β**_0_ = **log**(**Aff**_**R**_) + **log**(**t**_1**N**_ + **t**_2**N**_)	No special interest
${\boldsymbol{\upbeta}}_1=\mathbf{log}\left(\frac{\mathbf{Af}{\mathbf{f}}_1}{\mathbf{Af}{\mathbf{f}}_{\mathbf{R}}}\right)+\mathbf{log}\left(\frac{{\mathbf{t}}_{1\mathbf{N}}}{{\mathbf{t}}_{1\mathbf{N}}+{\mathbf{t}}_{2\mathbf{N}}}\right)$	No special interest
${\boldsymbol{\upbeta}}_2=\mathbf{log}\left(\frac{\mathbf{Af}{\mathbf{f}}_2}{\mathbf{Af}{\mathbf{f}}_1}\right)+\mathbf{log}\left(\frac{{\mathbf{t}}_{2\mathbf{N}}}{{\mathbf{t}}_{1\mathbf{N}}}\right)$	No special interest
${\boldsymbol{\upbeta}}_3=\mathbf{log}\left(\frac{{\mathbf{t}}_{1\mathbf{T}}+{\mathbf{t}}_{2\mathbf{T}}}{{\mathbf{t}}_{1\mathbf{N}}+{\mathbf{t}}_{2\mathbf{N}}}\right)$	Logarithm of the overall fold change of the event. Differential expression present if different from zero.
${\boldsymbol{\upbeta}}_4=\mathbf{log}\left(\frac{{\mathbf{t}}_{1\mathbf{T}}}{{\mathbf{t}}_{1\mathbf{T}}+{\mathbf{t}}_{2\mathbf{T}}}\right)-\mathbf{log}\left(\frac{{\mathbf{t}}_{1\mathbf{N}}}{{\mathbf{t}}_{1\mathbf{N}}+{\mathbf{t}}_{2\mathbf{N}}}\right)$	Difference of the logarithms of the fold change using relative concentrations of isoform 1 in both conditions. AS present if different from 0.
${\boldsymbol{\upbeta}}_5=\mathbf{log}\left(\frac{{\mathbf{t}}_{2\mathbf{T}}}{{\mathbf{t}}_{1\mathbf{T}}}\right)-\mathbf{log}\left(\frac{{\mathbf{t}}_{2\mathbf{N}}}{{\mathbf{t}}_{1\mathbf{N}}}\right)$	Difference of the logarithms of the fold change of both isoforms. AS present if different from zero.
${\boldsymbol{\upbeta}}_4+{\boldsymbol{\upbeta}}_5=\mathbf{log}\left(\frac{{\mathbf{t}}_{2\mathbf{T}}}{{\mathbf{t}}_{1\mathbf{T}}+{\mathbf{t}}_{2\mathbf{T}}}\right)-\mathbf{log}\left(\frac{{\mathbf{t}}_{2\mathbf{N}}}{{\mathbf{t}}_{1\mathbf{N}}+{\mathbf{t}}_{2\mathbf{N}}}\right)$	Difference of the logarithms of the fold change using relative concentrations of isoform 2 in both conditions. AS present if different from 0.
${\boldsymbol{\upbeta}}_3+{\boldsymbol{\upbeta}}_4 = \mathbf{log}\left(\frac{{\mathbf{t}}_{1\mathbf{T}}}{{\mathbf{t}}_{1\mathbf{N}}}\right)$	Logarithm of the fold change of isoform 1.
${\boldsymbol{\upbeta}}_3+{\boldsymbol{\upbeta}}_4+{\boldsymbol{\upbeta}}_5=\mathbf{log}\left(\frac{{\mathbf{t}}_{2\mathbf{T}}}{{\mathbf{t}}_{2\mathbf{N}}}\right)$	Logarithm of the fold change of isoform 2.

Within Table 4, there are some coefficients that involve affinities and, therefore, have little interest from a biological point of view. The first 6 rows provide the values of the *β*_*0*_ to *β*_*5*_. Rows 8 to 9 provide the values of linear combinations that have interest to detect splicing.

There are a number of alternatives to detect AS using these coefficients. Either of *β*_*4*_, *β*_*5*_, *β*_*4*_+ *β*_*5*_ is theoretically able to detect AS events. Some of them are more sensitive than others depending on the relative concentrations of the isoforms. For example, if isoform 2 is much more highly expressed than isoform 1 in both conditions, *β*_*4*_ will be more sensitive than *β*_*4*_+ *β*_*5*_ since in the latter case, the numerator and denominator of the logarithms of both terms are similar, and hence their logs are close to zero. A contrast on *β*_*5*_ would seem to be more sensitive than a contrast on *β*_*4*_ or *β*_*4*_+ *β*_*5*_; however, in practice, this contrast proved to be “too sensitive” and led to many false positives especially in weakly expressed isoforms. If one of the paths is not expressed in any condition, its signal will be similar in either condition (the background level) and a change in the expression of the other isoform will drive to a false positive detection. This contrast can be used only if the signals are filtered to guarantee that they are above the background.

In the PCR validation, the contrast that provided the best performance was the combination of the fold changes of both isoforms (i.e. *β*_*3*_ + *β*_*4*_ and *β*_*3*_ + *β*_*4*_ + *β*_*5*_ in Table 4) plus the requirement that the fold-changes have opposite directions, i.e. if isoform 1 significantly increases its expression, isoform 2 must significantly decrease its expression and visa versa. Therefore, if this test requires that the detected AS events show a significant change of the expression both paths and this change must be in opposite direction.

In order to compute this contrast, we summed up the p-values (one-tailed) for both contrasts. If the null hypothesis holds, the expected null distribution is triangular from 0 to 2 with the peak at 1, and the summation of the p-values must be close to 0 or close to 2 for genes with differential AS. Using this triangular distribution, it is possible to assign an overall p-value to their sum. We preferred this combination rather than the classical Fisher method since in the latter a single good p-value yields a good summary p-value for the event. Using this approach, both p-values must be close to zero or one in order to generate a significant overall p-value.

All the statistics had been implemented in an R package (available at Github), which depends on the limma method to get the statistical significance. Given the “standard” design matrix, **D** and the corresponding contrast matrix **C**, the software internally computes the design and contrast matrices of the events **D**_*DRS*_ and **C**_*DRS*_. Moreover, given a contrast matrix for the experiment, the contrast matrix to detect the splicing events is given by:

7 $$ {\mathbf{C}}_{DRS}=\left[\begin{array}{c}\hfill C\otimes \left[\begin{array}{ccc}\hfill 1\hfill & \hfill 1\hfill & \hfill 0\hfill \end{array}\right]\hfill \\ {}\hfill C\otimes \left[\begin{array}{ccc}\hfill 1\hfill & \hfill 1\hfill & \hfill 1\hfill \end{array}\right]\hfill \end{array}\right], $$

in where each row represents the constrasts β3+β4 and β3+β4+ β5 i.e. for each given contrast, the differential usage of both pathways is tested and summarized.

By construction, each contrast is split into two different contrasts (to test the differential expression of both isoforms) and afterwards, they are summarized and returned to the user.

#### Expression filter

The contrast previously described is very sensitive. If one of the paths of an event is not expressed and the other one is, EventPointer would assign a significant p-value to the event. This would result in a large number of false positives due to the lack of expression.

In order to avoid this problem, EventPointer allows the user to filter the events to ensure that all the paths are expressed above a fixed threshold. For every path, the algorithm gets the maximum value of expression from all the samples. The maximum values for the references are used to set the threshold. The user provides a quantile that will set the threshold.

Once the threshold is selected, an event will be considered as expressed if the maximum value of expression for all the paths is above the threshold previously set.

#### Domain expression analysis

Using the Ensembl database is possible to relate each of the paths P1 and P2 in the events with the presence of protein domains within them. For each domain, it is possible to know in which paths P1 and P2 is included. In order to state the statistical significance of the events, we performed a Wilcoxon test paired for each event (P1 and P2). This algorithm is done using sparse matrices and turns to be very efficient (less than 1/10th of second for all the domains in the Pfam database).

## Abbreviations

AS, alternative splicing; ASPIRE, analysis of splicing by isoform reprocity; ATCC, American type culture collection; EP, eventpointer; HJAY, human junction array; HTA, human transcriptome array; SI, splicing index; SS, splicing score; TAC, transcriptome analysis console

## Additional files

Additional file 1: Vignette of the use of the aroma.affymetrix framework to perform a gene expression analysis (SRSF1 knock-down analysis) with HTA 2.0 data. (PDF 300 kb) Additional file 2: Vignette of the use of the EventPointer on the study of SRSF1 using HTA 2.0 data. (PDF 246 kb) Additional file 3: PCR images and relative concentrations of the isoforms based on the PCR image. The red bar corresponds to the shorter isoform and the blue bar to the longer isoform. For the event in KIAA0100, it was not possible to get PCR results, and thus the image is not included. (TIF 1465 kb) Additional file 4: Table with primers used for validation and sizes of the expected splicing variants. (DOCX 24 kb) Additional file 5: Results for top-ranked events according to TAC 3.0. (DOCX 17 kb) Additional file 6: PCR images and relative concentrations of the NAA50 gene detected by TAC 3.0. The red bar corresponds to the shorter isoform and the blue bar to the longer isoform. (PNG 166 kb) Additional file 7: PCR images and relative concentrations of the isoforms based on the PCR image of gene DNM2. The grey bar corresponds to the shorter isoform and the white bar to the longer isoform. (JPG 92 kb) Additional file 8: Supplementary methods. Detection and classification of events. (DOCX 63 kb)
