# Supplementary material for: EventPointer: an effective identification of alternative splicing events using junction arrays
Source: BMC Genomics. 2016 Jun 17;17:467. doi: 10.1186/s12864-016-2816-x (PMC4912780; doi:10.1186/s12864-016-2816-x)
Supplement: Additional file 1: — Vignette of the use of the aroma.affymetrix framework to perform a gene expression analysis (SRSF1 knock-down analysis) with HTA 2.0 data. (PDF 300 kb) [file 12864_2016_2816_MOESM1_ESM.pdf]

# EventPointer: An effective identification of alternative splicing events using junction microarrays. Expression Analysis

EventPointer R package can be applied to complex experimental designs by giving the required contrast and design matrices. This vignette illustrates how how use aroma.affymetrix to perform the preprocessing steps on HTA2.0 arrays and how to exploit them. Gene expression is a complementary analysis to alternative splciing analysis. This vignette is therefore completely independent of EventPointer and is shown to illustrate the usage of aroma.affymetrix with HTA2 arrays.

## Table of Contents


- Introduction
- Example
- Dependencies
- Authors


## Introduction

EventPointer is a R package used to identify alternative splicing events in complex experimental designs, such as time course studies, paired samples or any other. The algorithm just requires the corresponding `design` and `contrast` matrices to be used for the experiment.

The algorithm tests all the identifiable events by the Affymetrix arrays: Human Transcriptome Array 2.0 (HTA 2.0) & Human Junction Array (Hjay). Each event is statistically tested to identify if the most expressed isoform changes between different conditions. This vignette is **not** related with the detection of Alternative splicing events but on how to use the aroma.affymetrix package on these arrays.

## Example

Analysis of the expression data.First of all is necessary to download a CDF file form Brianarray. Download custom cdfs for Ensembl from http://brainarray.mbni.med.umich.edu/Brainarray/Database/CustomCDF/CDF_download.asp

Go to last version 19 of the cdf files (version 20 seems to have a bug when working with aroma.affymetrix). Click on ENSG versions and go to Homo_sapiens and HTA20 (Affymetrix HTA20 array) row, click on the last option CDF/Seq/Map/Desc to download each file and extract the cdf files from zip compresed files

Brainarray cdf files are in ascii format. They should be converted into binary format to be used with aroma.affymetrix using the convertCdf command (It will take time, about 20 minutes). This conversion should be done only once.

```
library(knitr)
# EventPointer: Alternative splicing analysis.

########################################
# Aroma Affymetrix Preprocessing Pipeline
########################################
```

```
# Standard preprocessing pipeline for the microarray data
# functions and parameters are predefined according to
# the aroma.affymetrix R package

# Conversion of the CDF from Brainarray to binary file. Needs to be done only once.
library(aroma.affymetrix)
setOption(aromaSettings, "memory/ram", 8)

# UNCOMMENT THESE TWO LINES TO PERFORM THE CONVERSION

#setwd("~/../aroma.affymetrix/annotationData/chipTypes/HTA20")
#convertCdf("hta20_Hs_ENSG.cdf", "HTA-2_0,r_ENSG,brainarray,v19.cdf")

# CONVERSION DONE
# Done
```

It is necesssary to have a directory structure as explained in,

http://www.aroma-project.org/setup/QuickSummaryOfRequiredFileStructure/

Once the structure is properly set, this code should run without errors. It performs the background removal, quantile normalization and summarization for all the arrays in a experiment.

```
# Preprocess the samples
#setwd("~/../aroma.affymetrix") # Include here your directory.
setwd("/Volumes/Seagate_4/aroma.affymetrix")
verbose <- Arguments$getVerbose(-8);
future::plan("multiprocess") # Set the multiprocess to run faster
timestampOn(verbose);
projectName <- "SRSF1"
chipType <- "HTA-2_0"
cdfGFile <- "HTA-2_0,r_ENSG,brainarray,v19"
cdfG <- AffymetrixCdfFile$byChipType(cdfGFile)
cs <- AffymetrixCelSet$byName(projectName, cdf=cdfG)
bc <- NormExpBackgroundCorrection(cs, method="mle", tag=c("*","r11"));
csBC <- process(bc,verbose=verbose,ram=8);
qn <- QuantileNormalization(csBC, typesToUpdate="pm");
csN <- process(qn,verbose=verbose,ram=8);
plmEx <- ExonRmaPlm(csN, mergeGroups=FALSE)
fit(plmEx, verbose=verbose, ram = 8)
```

```
## 1,
```

```
cesEx <- getChipEffectSet(plmEx, ram = 8)
ExFit <- extractDataFrame(cesEx, addNames=TRUE)
# Done.

# Some processing to the data
# remove the _at tail from the names given in Brainarray.
ExFit$unitName <- unlist(strsplit(ExFit$unitName,"_"))[c(TRUE, FALSE)]
rownames(ExFit) <- ExFit$unitName
```

In ExFit we have a data.frame with the expression for all the genes in the array. Now, using limma is possible to get the differentially expressed genes.

```r
# Differential analysis of the samples
library(biomaRt)
library(limma)
library(knitr)

# Set Design and Contrast matrices
samplenames <- unlist(strsplit(colnames(ExFit)[6:ncol(ExFit)],"HTA2_", fixed = TRUE))[c(FALSE,TRUE)]
dummy <- unlist(strsplit(samplenames, "_"))[c(FALSE,TRUE,FALSE,FALSE)]
dfDesign <- data.frame(dummy)
colnames(dfDesign) <- c("Treat")
dfDesign$Treat <- relevel(dfDesign$Treat, ref = "LF")

Design <- model.matrix(~ ., data =  dfDesign)
Contrast <- c(0,-1,1)
```

Desing and Contrast Matrices

```r
Design
```

```
##    (Intercept) TreatSCR TreatsiSRSF1
## 1            1        0            0
## 2            1        0            0
## 3            1        0            0
## 4            1        0            0
## 5            1        0            0
## 6            1        0            0
## 7            1        0            0
## 8            1        0            0
## 9            1        0            0
## 10           1        1            0
## 11           1        1            0
## 12           1        1            0
## 13           1        1            0
## 14           1        1            0
## 15           1        1            0
## 16           1        1            0
## 17           1        1            0
## 18           1        1            0
## 19           1        0            1
## 20           1        0            1
## 21           1        0            1
## 22           1        0            1
## 23           1        0            1
## 24           1        0            1
## 25           1        0            1
## 26           1        0            1
## 27           1        0            1
## attr(,"assign")
## [1] 0 1 1
## attr(,"contrasts")
## attr(,"contrasts")$Treat
## [1] "contr.treatment"
```

```
Contrast
```

```
## [1]  0 -1  1
```

Perform the statistical analysis using limma

```
# Run limma
Y <- log2(as.matrix(ExFit[,6:ncol(ExFit)]))
colnames(Y) <- unlist(strsplit(colnames(Y),"HTA2_"))[c(FALSE,TRUE)]
fit <- lmFit(Y,design=Design)
fit2 <- contrasts.fit(fit, Contrast)
fit2 <- eBayes(fit2)
opts_chunk$set(fig.width=8, fig.height=6)
```

We can show some of the results:

```
opts_chunk$set(fig.width=8, fig.height=6)
# Obtain the ranking of events for each of the contrasts
Top10 <- rownames(topTable(fit2,coef=1,number=10))
#colorder <- order(Design %*% c(1,2,3,10) + .001 *(1:30))
library("RColorBrewer")
colfunc <- colorRampPalette(c("blue", "white", "red"))
heatmap(Y[Top10, ],col=colfunc(256))
```

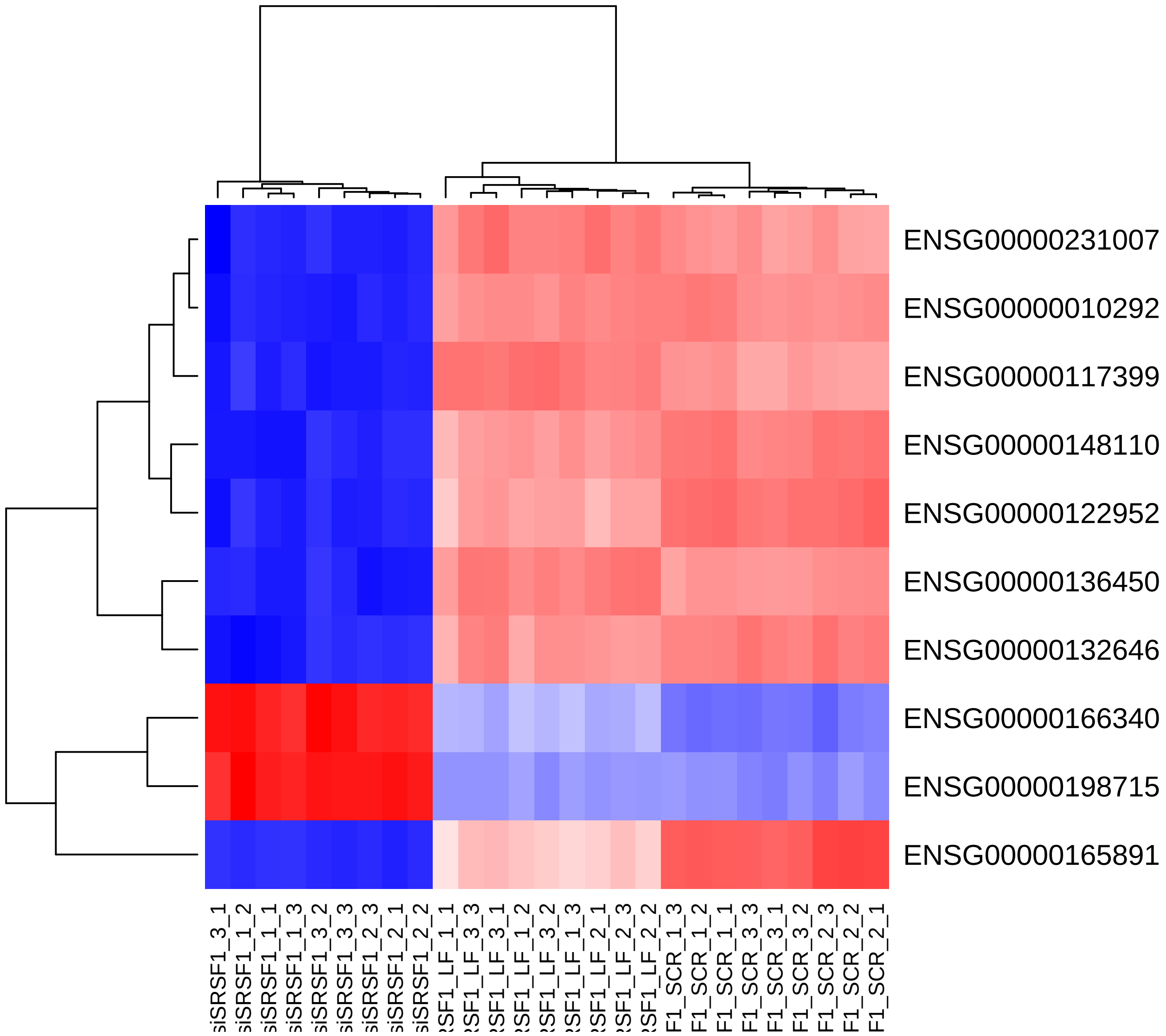


The HTA 20 array has many non-coding genes. The annotation of biotype from Ensembl can be downloaded and used with the following code:

```
opts_chunk$set(fig.width=8, fig.height=6)

# Use biomaRt to identify which genes in the array are lncRNA
ensembl <- useMart("hsapiens_gene_ensembl",
                   host="oct2014.archive.ensembl.org",
                   biomart="ENSEMBL_MART_ENSEMBL") #Get the hg19 version in biomart
genes <- getBM(attributes=c('ensembl_gene_id','strand',"chromosome_name",
                            "external_gene_name", "gene_biotype"),
               filters = "ensembl_gene_id",
               values = rownames(Y),
               mart = ensembl) # Oct2014 is the Ensembl version of Brainarray 19

# Resort genes according tothe expression matrix (Y)
rownames(genes) <- genes$ensembl_gene_id
genes <- genes[match(rownames(Y),genes$ensembl_gene_id),]
colnames(genes)[c(3,4)] <- c("chr","gene_name")
```

We can compare the expression of the genes in different biotypes:

```
# Select one sample and compare the expression according to the biotype
library(ggplot2)
biotype <- factor(genes$gene_biotype)
biotype <- relevel(biotype, "protein_coding")
qplot(biotype, Y[,1], geom="boxplot")+ coord_flip()
```

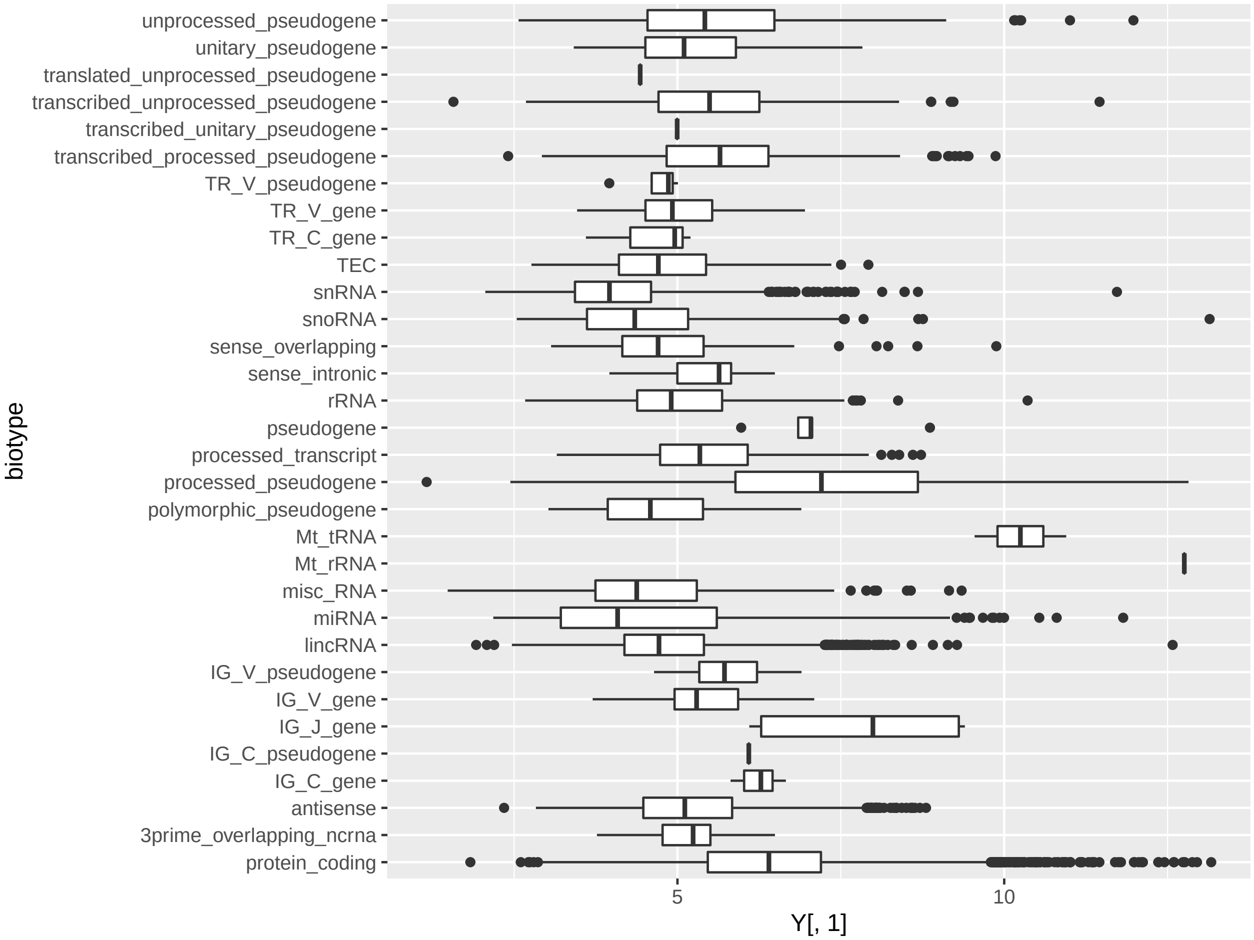


These values are shown for the first sample (WT) in the experiment. A similar result could be obtained ploting a different sample. For completeness, we show here the plot for a sample in which SRSF1 is knocked down.

```
qplot(biotype, Y[,19], geom="boxplot")+ coord_flip()
```

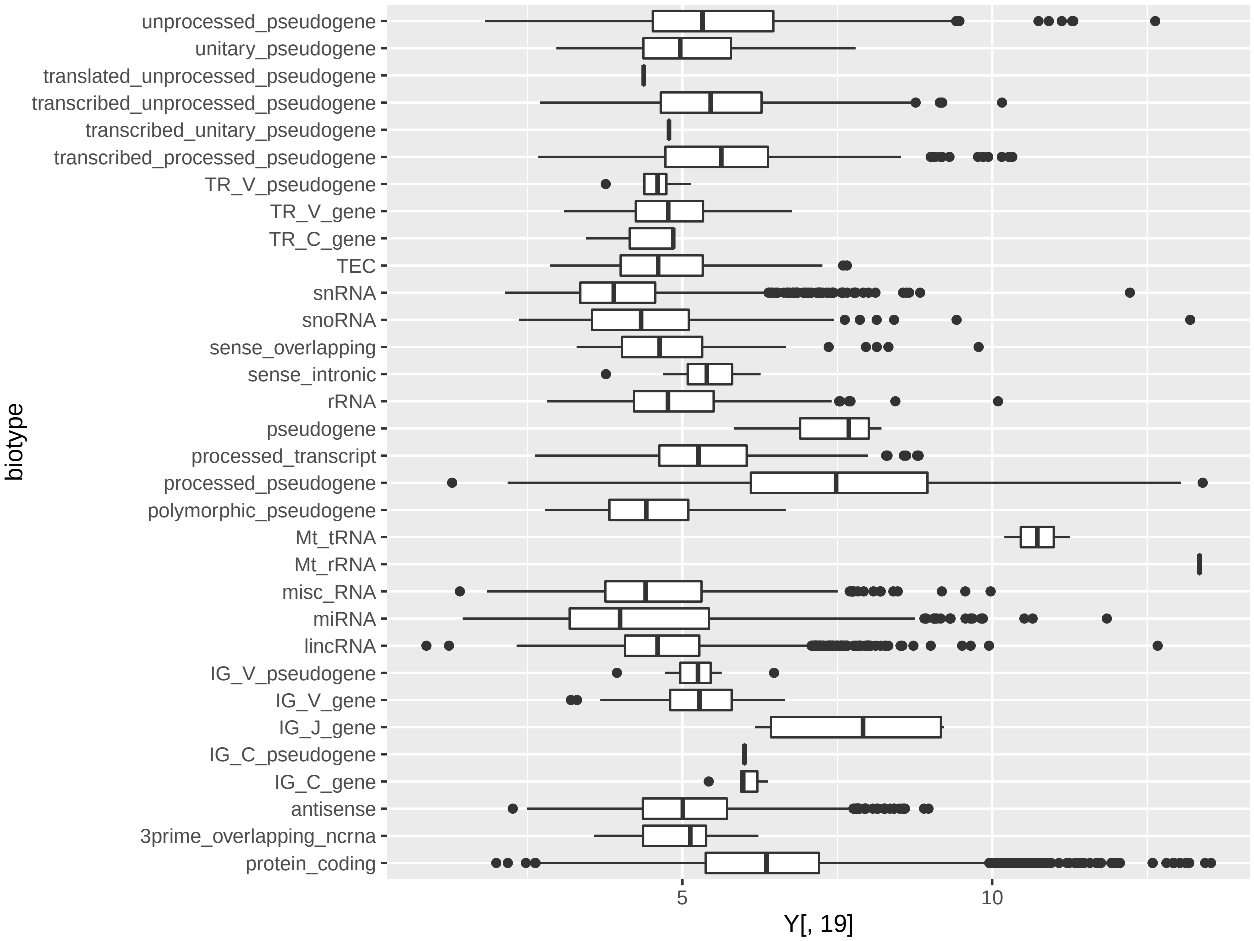


The meaning of each of the classes are explained in http://www.gencodegenes.org/gencode_biotypes.html and in http://uswest.ensembl.org/info/genome/genebuild/ncrna.html

Protein codeing genes (last row) are well expressed. Few biotypes are more expressed than them. We can use a linear model to test if any of the biotypes are more expressed than the protein coding genes.

```
# Protein coding are more expressed than others (except a few biotypes)
# Most of the coefficients are negative

Salida <- summary(lm(Y[,1]~biotype))$coefficients
```

The only positive significant subclasses are:

```
knitr::kable(Salida[ which(Salida[,1]>0 & Salida[,4] < 0.05),])
```

| | Estimate | Std. Error | t value | Pr(>\|t\|) |
|---|---|---|---|---|
| (Intercept) | 6.3548807 | 0.0090514 | 702.087786 | 0.0000000 |
| biotypeIG_J_gene | 1.4673021 | 0.4991831 | 2.939407 | 0.0032905 |
| biotypeMt_rRNA | 6.4012581 | 1.2225764 | 5.235876 | 0.0000002 |
| biotypeMt_tRNA | 3.8932128 | 0.8645158 | 4.503345 | 0.0000067 |
| biotypeprocessed_pseudogene | 0.9662092 | 0.0315428 | 30.631717 | 0.0000000 |

The following code shows a list of the top differentially expressed linc genes

```
lincgenes <- which(genes$gene_biotype == "lincRNA")
Ylinc <- Y[lincgenes,]
fit <- lmFit(Ylinc,design=Design)
fitlinc <- contrasts.fit(fit, Contrast)
fitlinc <- eBayes(fitlinc)
```

```
knitr::kable(topTable(fitlinc,coef=1,number=10,genes[lincgenes,c(1,4)])[,-1])
```

| | gene_name | logFC | AveExpr | t | P.Value | adj.P.Val | B |
|---|---|---|---|---|---|---|---|
| ENSG00000250271 | RP11-64D22.5 | 2.4317325 | 7.907102 | 24.07660 | 0 | 0 | 38.23847 |
| ENSG00000225434 | LINC01504 | 0.8423124 | 6.228831 | 22.07674 | 0 | 0 | 35.91971 |
| ENSG00000272275 | RP11-791G15.2 | 1.1073668 | 6.629803 | 21.64433 | 0 | 0 | 35.38885 |
| ENSG00000256268 | RP11-221N13.3 | 1.1328192 | 5.089143 | 20.61771 | 0 | 0 | 34.08293 |
| ENSG00000272016 | RP11-215G15.5 | -1.5614063 | 8.140296 | -19.22318 | 0 | 0 | 32.19901 |
| ENSG00000187185 | CTD-2600O9.1 | 0.8415443 | 7.079163 | 17.79792 | 0 | 0 | 30.12960 |
| ENSG00000235823 | LINC00263 | -0.8076224 | 5.763845 | -17.18215 | 0 | 0 | 29.18646 |
| ENSG00000260804 | PKI55 | 0.7826596 | 6.178655 | 16.51176 | 0 | 0 | 28.12355 |
| ENSG00000235609 | AF127936.7 | 0.6657937 | 4.857875 | 16.17517 | 0 | 0 | 27.57510 |
| ENSG00000258815 | RP11-408B11.2 | 1.3493025 | 4.216329 | 15.80909 | 0 | 0 | 26.96695 |

```
Top10 <- rownames(topTable(fitlinc,coef=1,number=10,genes[lincgenes,c(4)]))
colfunc <- colorRampPalette(c("blue", "white", "red"))
```

and plots the heatmap

```
heatmap(Ylinc[Top10, ],col=colfunc(256))
```

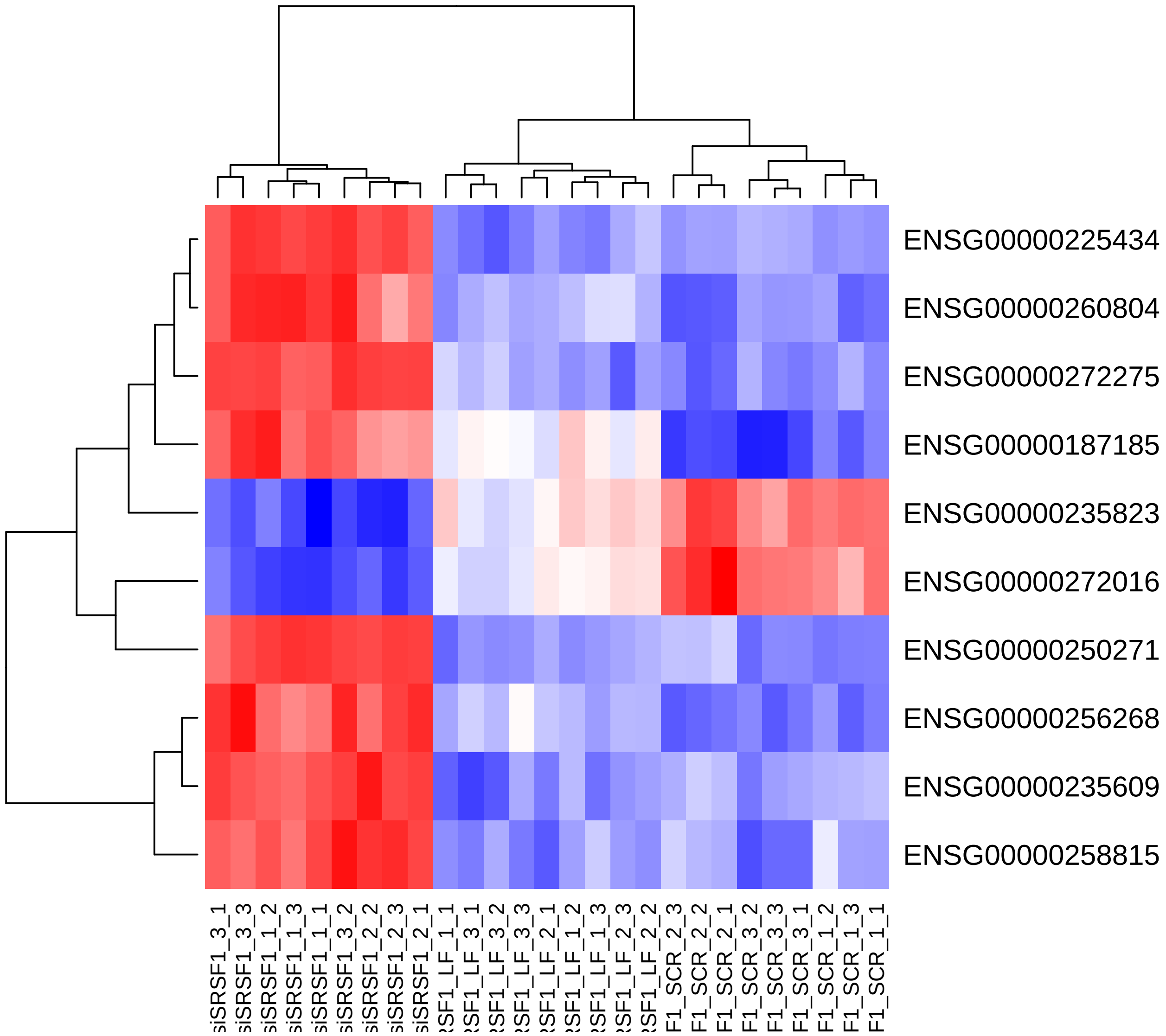


```
ggplot(as.data.frame.table(t(Ylinc[Top10,])), aes(x = Var1, y = Freq, group = Var2, colour = Var2)) +
geom_point() +
geom_line(aes(lty = Var2))+ theme(axis.text.x = element_text(angle = 90, hjust = 1))
```

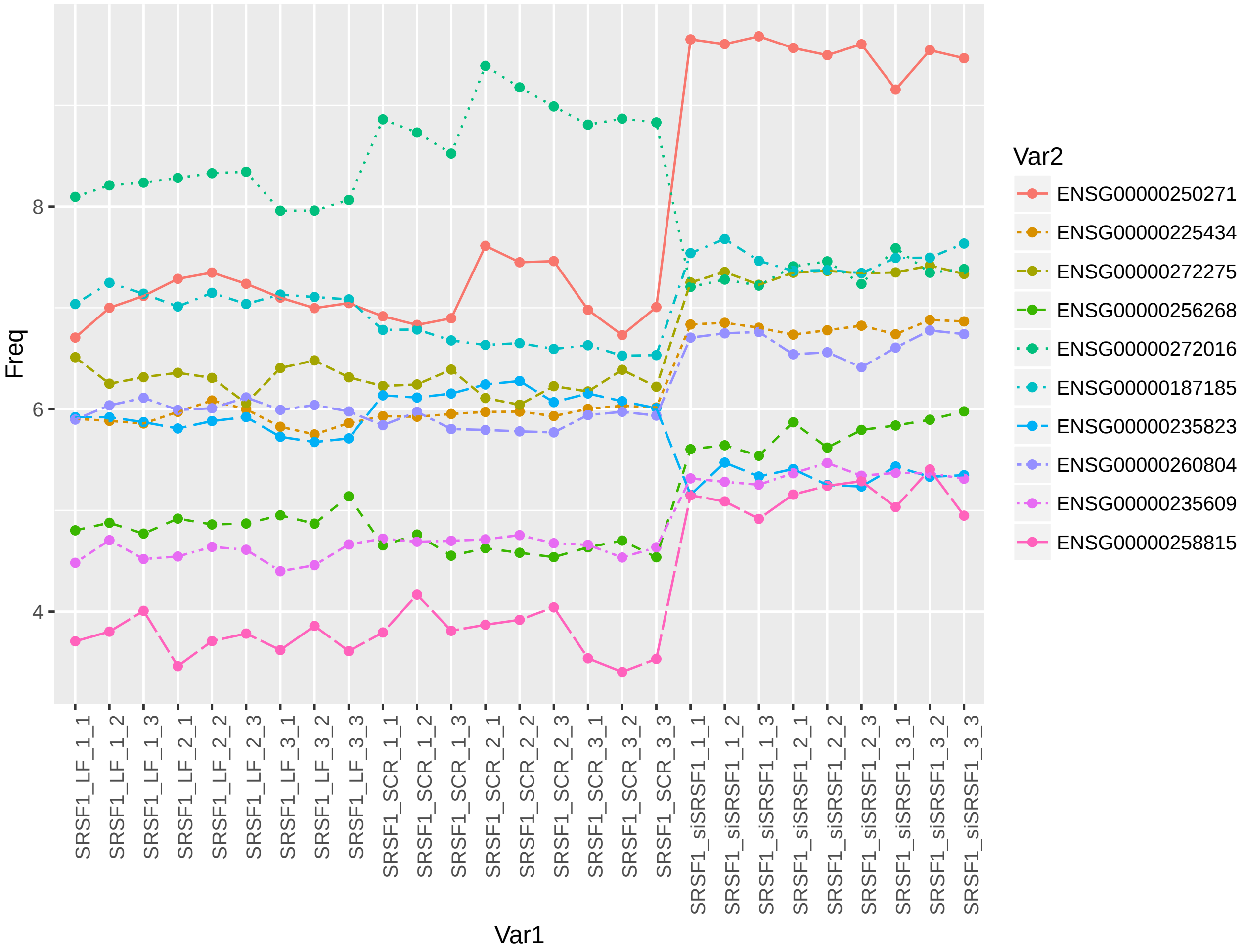


MALAT1 (ENSG00000251562), is known to be related by SRSF1 (http://www.sciencedirect.com/science/article/pii/S2211124712001635). However, in this specific experiment, did not show a local false discovery rate bigger than 0.5 (i.e. the B column of the limma analysis positive). Nevertheless, the adjusted p.value is significant (< .05).

```
Output <- topTable(fitlinc,coef=1,number=Inf,genes[lincgenes,c(1,4)])
knitr::kable(Output[match("MALAT1", Output$gene_name),-1])
```

| | gene_name | logFC | AveExpr | t | P.Value | adj.P.Val | B |
|---|---|---|---|---|---|---|---|
| ENSG00000251562 | MALAT1 | 0.2627201 | 9.551345 | 3.931334 | 0.0004533 | 0.0112304 | -0.7527798 |

PDXDC2P, GHRLOS ncRNAs have also been related with SRSF1. They are not annotated as lincRNA but as processed transcript and antisense respectively. The p.value of GHRLOS is significant but the fold change (the effect size) is small.

```
Output <- topTable(fit2,coef=1,number=Inf,genes[,c(1,4)])
knitr::kable(Output[genes[match(c("PDXDC2P","GHRLOS"), genes$gene_name),1],-1])
```

| | gene_name | logFC | AveExpr | t | P.Value | adj.P.Val | B |
|---|---|---|---|---|---|---|---|
| ENSG00000196696 | PDXDC2P | 0.4379363 | 6.018468 | 2.237197 | 0.0336259 | 0.0899362 | -5.6778838 |

| | gene_name | logFC | AveExpr | t | P.Value | adj.P.Val | B |
|---|---|---|---|---|---|---|---|
| ENSG00000240288 | GHRLOS | -0.1990165 | 5.513184 | -4.727033 | 0.0000618 | 0.0002984 | 0.3940236 |

## Gene Ontology Analysis

It is possible to run an enrichment analysis with the data.

```
# GO enrichment analysis
library(org.Hs.eg.db)
library(topGO)

# Overexpressed
GOdata <- new("topGOdata", ontology = "BP", allGenes = fit2$t[,1], geneSel = function(t) t  > 3,
              description = "Test", annot = annFUN.org, mapping = "org.Hs.eg.db",
              ID = "Ensembl")
resultFisher <- runTest(GOdata, algorithm = "weight01", statistic = "fisher")
```

The result is

```
knitr::kable(GenTable(GOdata, wFisher = resultFisher, topNodes = 20))
```

| GO.ID | Term | Annotated | Significant | Expected | wFisher |
|---|---|---|---|---|---|
| GO:0006614 | SRP-dependent cotranslational protein ta... | 105 | 67 | 23.84 | 2.0e-19 |
| GO:0006414 | translational elongation | 198 | 77 | 44.95 | 2.8e-17 |
| GO:0016259 | selenocysteine metabolic process | 87 | 57 | 19.75 | 4.5e-17 |
| GO:0000184 | nuclear-transcribed mRNA catabolic proce... | 116 | 66 | 26.34 | 3.2e-15 |
| GO:0006415 | translational termination | 171 | 65 | 38.82 | 5.8e-15 |
| GO:0006413 | translational initiation | 256 | 96 | 58.12 | 2.3e-13 |
| GO:0019083 | viral transcription | 187 | 79 | 42.45 | 2.9e-10 |
| GO:0018279 | protein N-linked glycosylation via aspar... | 245 | 98 | 55.62 | 7.6e-10 |
| GO:0035666 | TRIF-dependent toll-like receptor signal... | 81 | 38 | 18.39 | 1.3e-06 |
| GO:0045454 | cell redox homeostasis | 87 | 40 | 19.75 | 1.3e-06 |
| GO:0006355 | regulation of transcription, DNA-templat... | 3319 | 863 | 753.52 | 2.4e-06 |
| GO:0034138 | toll-like receptor 3 signaling pathway | 95 | 42 | 21.57 | 2.7e-06 |
| GO:0034142 | toll-like receptor 4 signaling pathway | 115 | 47 | 26.11 | 6.9e-06 |
| GO:0036498 | IRE1-mediated unfolded protein response | 64 | 30 | 14.53 | 9.4e-06 |
| GO:0006367 | transcription initiation from RNA polyme... | 268 | 88 | 60.84 | 1.5e-05 |
| GO:0034134 | toll-like receptor 2 signaling pathway | 82 | 35 | 18.62 | 2.4e-05 |
| GO:0034162 | toll-like receptor 9 signaling pathway | 77 | 33 | 17.48 | 6.5e-05 |
| GO:0038123 | toll-like receptor TLR1:TLR2 signaling p... | 75 | 32 | 17.03 | 9.2e-05 |
| GO:0038124 | toll-like receptor TLR6:TLR2 signaling p... | 75 | 32 | 17.03 | 9.2e-05 |
| GO:0034146 | toll-like receptor 5 signaling pathway | 70 | 31 | 15.89 | 9.9e-05 |

```
# Underexpressed
GOdata@geneSelectionFun <- function(t) t  < -3
resultFisher <- runTest(GOdata, algorithm = "weight01", statistic = "fisher")
```

and

```r
knitr::kable(GenTable(GOdata, wFisher = resultFisher, topNodes = 20))
```

| GO.ID | Term | Annotated | Significant | Expected | wFisher |
|---|---|---|---|---|---|
| GO:0051301 | cell division | 628 | 282 | 144.50 | 1.6e-27 |
| GO:0007067 | mitotic nuclear division | 414 | 223 | 95.26 | 1.9e-19 |
| GO:0000724 | double-strand break repair via homologou... | 145 | 94 | 33.36 | 2.5e-17 |
| GO:0031047 | gene silencing by RNA | 141 | 72 | 32.44 | 4.5e-16 |
| GO:0000086 | G2/M transition of mitotic cell cycle | 188 | 95 | 43.26 | 9.4e-16 |
| GO:0000082 | G1/S transition of mitotic cell cycle | 242 | 123 | 55.68 | 4.9e-15 |
| GO:0000398 | mRNA splicing, via spliceosome | 273 | 136 | 62.82 | 7.5e-15 |
| GO:0006271 | DNA strand elongation involved in DNA re... | 38 | 34 | 8.74 | 2.1e-14 |
| GO:0034080 | CENP-A containing nucleosome assembly | 38 | 31 | 8.74 | 3.3e-14 |
| GO:0016925 | protein sumoylation | 123 | 66 | 28.30 | 1.9e-13 |
| GO:0000278 | mitotic cell cycle | 1007 | 508 | 231.71 | 5.4e-13 |
| GO:0006303 | double-strand break repair via nonhomolo... | 62 | 42 | 14.27 | 8.0e-13 |
| GO:0006335 | DNA replication-dependent nucleosome ass... | 29 | 25 | 6.67 | 9.2e-13 |
| GO:0051290 | protein heterotetramerization | 35 | 28 | 8.05 | 1.5e-12 |
| GO:0031145 | anaphase-promoting complex-dependent pro... | 83 | 49 | 19.10 | 8.6e-12 |
| GO:0007264 | small GTPase mediated signal transductio... | 893 | 271 | 205.48 | 2.3e-11 |
| GO:0032508 | DNA duplex unwinding | 54 | 37 | 12.43 | 2.4e-11 |
| GO:0008033 | tRNA processing | 139 | 75 | 31.98 | 4.8e-11 |
| GO:0070125 | mitochondrial translational elongation | 79 | 45 | 18.18 | 7.6e-11 |
| GO:0007077 | mitotic nuclear envelope disassembly | 43 | 30 | 9.89 | 9.4e-11 |

One of the advantages of the topGO package is that avoids providing redundant information. For example, if a function is strongly enriched, also its parent in the ontology will probably be enriched. By using proper pruning, only the most significant leaves of the tree are provided. In some cases, a function expected to be affected does not appear simply because one of its descendant (and therefore more specific) functions are included in the output. The "classical" analysis can be performed by using a different algorithm that does not prune the significant functions. The results are in this case:

```r
# GO enrichment analysis
library(org.Hs.eg.db)
library(topGO)

# Overexpressed
GOdata <- new("topGOdata", ontology = "BP", allGenes = fit2$t[,1], geneSel = function(t) t  > 3,
              description = "Test", annot = annFUN.org, mapping = "org.Hs.eg.db",
              ID = "Ensembl")
resultFisher <- runTest(GOdata, algorithm = "classic", statistic = "fisher")
```

The result is

```r
knitr::kable(GenTable(GOdata, classicFisher = resultFisher, topNodes = 20))
```

| GO.ID | Term | Annotated | Significant | Expected | classicFisher |
|---|---|---|---|---|---|
| GO:0008152 | metabolic process | 11208 | 2763 | 2544.57 | 2.9e-21 |
| GO:0070972 | protein localization to endoplasmic reti... | 131 | 80 | 29.74 | 3.6e-21 |
| GO:0044260 | cellular macromolecule metabolic process | 8022 | 2057 | 1821.25 | 1.1e-19 |
| GO:0072599 | establishment of protein localization to... | 112 | 70 | 25.43 | 1.7e-19 |

| GO.ID | Term | Annotated | Significant | Expected | classicFisher |
|---|---|---|---|---|---|
| GO:0006614 | SRP-dependent cotranslational protein ta... | 105 | 67 | 23.84 | 2.0e-19 |
| GO:0045047 | protein targeting to ER | 108 | 68 | 24.52 | 3.1e-19 |
| GO:0034645 | cellular macromolecule biosynthetic proc... | 4775 | 1300 | 1084.08 | 5.3e-19 |
| GO:0044237 | cellular metabolic process | 9708 | 2426 | 2204.02 | 7.5e-19 |
| GO:0006613 | cotranslational protein targeting to mem... | 107 | 67 | 24.29 | 8.7e-19 |
| GO:0016259 | selenocysteine metabolic process | 87 | 57 | 19.75 | 1.6e-17 |
| GO:0009059 | macromolecule biosynthetic process | 4930 | 1327 | 1119.27 | 1.8e-17 |
| GO:0046907 | intracellular transport | 1812 | 557 | 411.38 | 2.3e-17 |
| GO:1902582 | single-organism intracellular transport | 1660 | 517 | 376.87 | 2.7e-17 |
| GO:0043170 | macromolecule metabolic process | 8636 | 2178 | 1960.64 | 3.2e-17 |
| GO:0044238 | primary metabolic process | 9764 | 2423 | 2216.74 | 1.5e-16 |
| GO:0071704 | organic substance metabolic process | 10065 | 2488 | 2285.07 | 1.9e-16 |
| GO:0009057 | macromolecule catabolic process | 1131 | 370 | 256.77 | 6.8e-16 |
| GO:0000184 | nuclear-transcribed mRNA catabolic proce... | 116 | 66 | 26.34 | 1.6e-15 |
| GO:0044248 | cellular catabolic process | 1653 | 504 | 375.28 | 5.6e-15 |
| GO:0044249 | cellular biosynthetic process | 5800 | 1513 | 1316.78 | 7.5e-15 |

```r
# Underexpressed
GOdata@geneSelectionFun <- function(t) t  < -3
resultFisher <- runTest(GOdata, algorithm = "classic", statistic = "fisher")
```

and

```r
knitr::kable(GenTable(GOdata, classicFisher = resultFisher, topNodes = 20))
```

| GO.ID | Term | Annotated | Significant | Expected | classicFisher |
|---|---|---|---|---|---|
| GO:0000278 | mitotic cell cycle | 1007 | 508 | 231.71 | <1e-30 |
| GO:0006996 | organelle organization | 3531 | 1251 | 812.48 | <1e-30 |
| GO:0007049 | cell cycle | 1738 | 735 | 399.91 | <1e-30 |
| GO:1903047 | mitotic cell cycle process | 838 | 431 | 192.82 | <1e-30 |
| GO:0022402 | cell cycle process | 1262 | 573 | 290.39 | <1e-30 |
| GO:0051276 | chromosome organization | 1058 | 469 | 243.45 | <1e-30 |
| GO:1902589 | single-organism organelle organization | 2544 | 892 | 585.38 | <1e-30 |
| GO:0034641 | cellular nitrogen compound metabolic pro... | 6144 | 1794 | 1413.74 | <1e-30 |
| GO:0006974 | cellular response to DNA damage stimulus | 766 | 353 | 176.26 | <1e-30 |
| GO:0044237 | cellular metabolic process | 9708 | 2596 | 2233.81 | <1e-30 |
| GO:0006807 | nitrogen compound metabolic process | 6435 | 1845 | 1480.70 | <1e-30 |
| GO:0006396 | RNA processing | 781 | 351 | 179.71 | <1e-30 |
| GO:0044772 | mitotic cell cycle phase transition | 469 | 244 | 107.92 | <1e-30 |
| GO:0071840 | cellular component organization or bioge... | 6030 | 1745 | 1387.51 | <1e-30 |
| GO:0044770 | cell cycle phase transition | 493 | 252 | 113.44 | <1e-30 |
| GO:0007067 | mitotic nuclear division | 414 | 223 | 95.26 | <1e-30 |
| GO:0000280 | nuclear division | 533 | 264 | 122.64 | <1e-30 |
| GO:0006281 | DNA repair | 486 | 245 | 111.83 | <1e-30 |
| GO:0044238 | primary metabolic process | 9764 | 2582 | 2246.70 | <1e-30 |
| GO:0006259 | DNA metabolic process | 929 | 391 | 213.76 | <1e-30 |

## Dependencies

- aroma.affymetrix Microarray pre-processing
- limma Statistical framework
- Matrix Sparse Matrices
- matrixStats Statistical functions on matrices
- dcGOR Protein Domains

## Session Info

```r
sessionInfo()
```

```
## R version 3.3.0 beta (2016-04-04 r70420)
## Platform: x86_64-apple-darwin13.4.0 (64-bit)
## Running under: OS X 10.11.4 (El Capitan)
##
## locale:
## [1] en_US.UTF-8/en_US.UTF-8/en_US.UTF-8/C/en_US.UTF-8/en_US.UTF-8
##
## attached base packages:
## [1] parallel  stats4    stats     graphics  grDevices utils     datasets
## [8] methods   base
##
## other attached packages:
##  [1] topGO_2.23.4           SparseM_1.7            GO.db_3.3.0
##  [4] graph_1.49.1           org.Hs.eg.db_3.3.0     AnnotationDbi_1.33.13
##  [7] IRanges_2.5.46         S4Vectors_0.9.52       Biobase_2.31.3
## [10] BiocGenerics_0.17.5    RColorBrewer_1.1-2     limma_3.27.19
## [13] biomaRt_2.27.2         aroma.light_3.1.1      aroma.affymetrix_3.0.0
## [16] aroma.core_3.0.0       R.devices_2.14.0       R.filesets_2.10.0
## [19] R.utils_2.3.0          R.oo_1.20.0            ggplot2_2.1.0
## [22] affxparser_1.43.2      R.methodsS3_1.7.1      knitr_1.12.3
## [25] BiocInstaller_1.21.4
##
## loaded via a namespace (and not attached):
##  [1] R.huge_0.9.0       DNAcopy_1.45.0     listenv_0.6.0
##  [4] lattice_0.20-33    colorspace_1.2-6   htmltools_0.3.5
##  [7] yaml_2.1.13        base64enc_0.1-4    XML_3.98-1.4
## [10] DBI_0.3.1          matrixStats_0.50.2 R.cache_0.12.0
## [13] plyr_1.8.3         stringr_1.0.0      munsell_0.4.3
## [16] gtable_0.2.0       future_0.13.0      codetools_0.2-14
## [19] evaluate_0.8.3     labeling_0.3       highr_0.5.1
## [22] Rcpp_0.12.4.5      scales_0.4.0       formatR_1.3
## [25] PSCBS_0.61.0       R.rsp_0.21.0       digest_0.6.9
## [28] stringi_1.0-1      grid_3.3.0         tools_3.3.0
## [31] bitops_1.0-6       magrittr_1.5       RCurl_1.95-4.8
## [34] RSQLite_1.0.0      rmarkdown_0.9.5    globals_0.6.1
## [37] aroma.apd_0.6.0
```

## Authors

Juan Pablo Romero (jpromero@ceit.es) : Bioinformatics Group, CEIT, San Sebastian, Spain

Angel Rubio : Bioinformatics Group, CEIT, San Sebastian, Spain
Ander Muniategui: Bioinformatics Group, CEIT, San Sebastian, Spain
