# Supplementary material for: EventPointer: an effective identification of alternative splicing events using junction arrays
Source: BMC Genomics. 2016 Jun 17;17:467. doi: 10.1186/s12864-016-2816-x (PMC4912780; doi:10.1186/s12864-016-2816-x)
Supplement: Additional file 2: — Vignette of the use of the EventPointer on the study of SRSF1 using HTA 2.0 data. (PDF 246 kb) [file 12864_2016_2816_MOESM2_ESM.pdf]

EventPointer: An effective identification of alternative splicing events using junction microarrays. Alternative Splicing

EventPointer R package can be applied to complex experimental designs by giving the required contrast and design matrices. This vignette illustrates how how use EventPointer to perform the analysis of alternative splicing in a experiment. The package aroma.affymetrix is used to perform the preprocessing steps on HTA2.0.

Table of Contents

- Introduction
- Example
- Dependencies
- Authors

Introduction

EventPointer is a R package used to identify alternative splicing events in complex experimental designs, such as time course studies, paired samples or any other. The algorithm just requires the corresponding `design` and `contrast` matrices to be used for the experiment.

The algorithm tests all the identifiable events by the Affymetrix arrays: Human Transcriptome Array 2.0 (HTA 2.0) & Human Junction Array (Hjay). Each event is statistically tested to identify if the most expressed isoform changes between different conditions.

Example

Analysis of the alternative splicing data. First of all is necessary to download a CDF file. It can be found at http://www.ncbi.nlm.nih.gov/geo/query/acc.cgi?acc=GPL21339

```
library(knitr)
# EventPointer: Alternative splicing analysis.

######################################
# Aroma Affymetrix Preprocessing Pipeline
######################################

# Standard preprocessing pipeline for the microarray data
# functions and parameters are predefined according to
# the aroma.affymetrix R package

library(aroma.affymetrix)
setOption(aromaSettings, "memory/ram", 8)
# Done
```

It is necesssary to have a directory structure as explained in,

http://www.aroma-project.org/setup/QuickSummaryOfRequiredFileStructure/

Once the structure is properly set, this code should run without errors. It performs the background removal, quantile normalization and summarization for all the arrays in a experiment.

```
# Preprocess the samples
#setwd("~/../aroma.affymetrix") # Include here your directory.
setwd("/Volumes/Seagate_4/aroma.affymetrix")
verbose <- Arguments$getVerbose(-8);
future::plan("multiprocess") # Set the multiprocess to run faster
timestampOn(verbose);
projectName <- "SRSF1-ASpaper"
chipType <- "HTA-2_0-ASpaper"
cdfGFile <- "HTA-2_0-ASpaper"
cdfG <- AffymetrixCdfFile$byChipType(cdfGFile)
cs <- AffymetrixCelSet$byName(projectName, cdf=cdfG)
bc <- NormExpBackgroundCorrection(cs, method="mle", tag=c("*","r11"));
csBC <- process(bc,verbose=verbose,ram=8);
qn <- QuantileNormalization(csBC, typesToUpdate="pm");
csN <- process(qn,verbose=verbose,ram=8);
plmEx <- ExonRmaPlm(csN, mergeGroups=FALSE)
fit(plmEx, verbose=verbose, ram = 8, force = TRUE)
```

```
## 1,
```

```
cesEx <- getChipEffectSet(plmEx, ram = 8)
ExFit <- extractDataFrame(cesEx, addNames=TRUE)
# Done.
```

In ExFit we have a data.frame with the expression for all the genes in the array. We build the contrast and design matrix using some characteristics of the names of the samples.

```
# Differential analysis of the samples
library(biomaRt)
library(limma)
library(knitr)
library(EventPointer)
library(ggplot2)

# Set Design and Contrast matrices
samplenames <- unlist(strsplit(colnames(ExFit)[6:ncol(ExFit)],"HTA2_", fixed = TRUE))[c(FALSE,TRUE)]
dfDesign <- data.frame(matrix(unlist(strsplit(samplenames, "_"))[c(FALSE,TRUE,FALSE,FALSE)], ncol =1, b
colnames(dfDesign) <- c("Treat")
dfDesign$Treat <- relevel(dfDesign$Treat, ref = "LF")

Design <- model.matrix(~ ., data =  dfDesign)
Contrast <- c(0,-1,1)
```

Now we are ready to run EventPointer.

```
##########################################
# Event Pointer
##########################################

# Alternative Splicing Analysis
Events<-EventPointer(Design=Design, Contrast=Contrast, affy=cesEx, array="HTA", Filter=T)
```

```
## 14:16:23  Running EventPointer
##  14:16:51  Analysis Completed
```

```r
opts_chunk$set(fig.width=8, fig.height=6)
```

The Top 10 differentially expressed genes are:

```r
Output <- Events[1:10,c(1,3,5,6)]
colnames(Output)[4] <- "Z.value(AS)"

knitr::kable(Output, digits=3)
```

	HGNC Symbol	Event Type	Genomic Position	Z.value(AS)
ENSG00000005810_7	MYCBP2	Complex Event	13:77673020-77695630	-16.703
ENSG00000137807_2	KIF23	Cassette Exon	15:69713986-69714774	16.158
ENSG00000214135_6		Cassette Exon	3:197348575-197350253	16.110
ENSG00000167196_2	FBXO22	Cassette Exon	15:76196323-76205608	14.968
ENSG00000112081_2	SRSF3	Complex Event	6:36566626-36568967	-14.741
ENSG00000092201_4	SUPT16H	Alternative Last Exon	14:21837979-21852105	14.603
ENSG00000147421_9	HMBOX1	Alternative First Exon	8:28902878-28904970	-13.235
ENSG00000240303_12	ACAD11	Complex Event	3:132297677-132298402	-12.989
ENSG00000141027_26	NCOR1	Cassette Exon	17:16052765-16055312	-12.882
ENSG00000115307_6	AUP1	Retained Intron	2:74754863-74755133	-12.526

Within the Events data.frame, one column is the (unadjusted) p.value. By construction, the p.values from genes that are differentially **bold** expressed **bold** are close to one, and for the ones differentially **bold** spliced **bold** are close to zero. For this reason the distribution of the p.values hace also a strong peak at 1. Multple diagnosis adjust must be done cautoiusly since the shape of the p.value histogram has this peak at zero. Even though the results may not be accurate, the adjst gives an idea on the overall modification of the splicing pattern. The corresponding code is:

```r
p.adjusted  <- p.adjust(Events$`Splicing Pvalue`[Events$`Splicing Pvalue`], method = "fdr")
```

The number of genes with alternative splicing (FDR < 0.05) is

```r
sum(p.adjusted < 0.05)
```

```
## [1] 315
```

Each event has three subregions of the gene assigned to them: the reference and two differential paths. For example, in the case of a cassette exon the reference are the flanking exons, and the two differential paths are the one that skips the exon (the probeset that corresponds to the junction) and the one that reatins the exons (the probesets that interrogate the exon itself and the juntions flanking it). The expressions of these paths can be shown. In this case we will do it for the event with the largest statistical significance.

```r
opts_chunk$set(fig.width=8, fig.height=6)
unit <- match(rownames(Events[1,]), ExFit$unitName)
Aux <- ExFit[unit:(unit+2),6:ncol(ExFit)]
colnames(Aux) <- samplenames
```

```
rownames(Aux) <- paste(ExFit[unit:(unit+2),1],ExFit[unit:(unit+2),2],sep="")

ggplot(as.data.frame.table(t(Aux)), aes(x = Var1, y = Freq, group = Var2, colour = Var2)) +
geom_point() +
geom_line(aes(lty = Var2))+ theme(axis.text.x = element_text(angle = 90, hjust = 1))
```

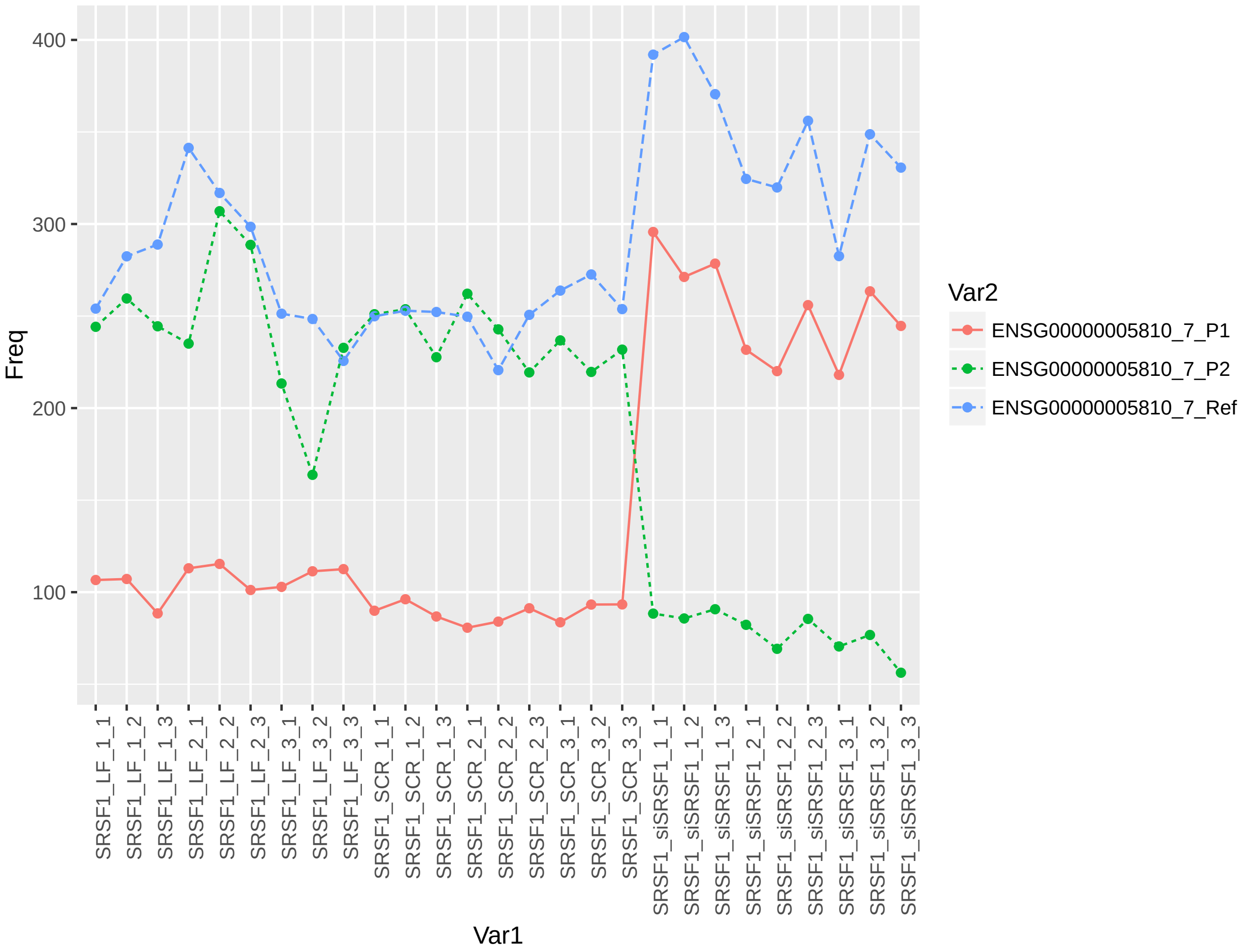

There is a change in the expression in this gene (the blue line that corresponds to the reference is more expressed). In addition and more importantly, there is a change in the usage of the exon: the red line (inclusion) is overexpressed when SRSFS1 is knocked out and the green line (exclusion) is downregulated.

Within EventPointer is also possible to check which are the PFAM domains that are altered because of the splicing (i.e. compute the enrichment of usage of the domains correcting for the expression)

```
# Domain Enrichments
Domains<-DomainEnrichment(Events=Events)
```

The results can be shown by:

Upregulated Domains

```r
# Domain Enrichments
knitr::kable(Domains$UpRegulated_Domain)
```

	Domain	Pvalue	Zvalue	Info
PF00052	PF00052	0.0e+00	7.556239	Laminin B (Domain IV)
PF08373	PF08373	0.0e+00	6.231828	RAP domain
PF00400	PF00400	0.0e+00	6.081891	WD domain, G-beta repeat
PF06008	PF06008	0.0e+00	6.036628	Laminin Domain I
PF06009	PF06009	0.0e+00	5.986839	Laminin Domain II
PF02780	PF02780	0.0e+00	5.974939	Transketolase, C-terminal domain
PF04836	PF04836	0.0e+00	5.969589	Interferon-related protein conserved region
PF05004	PF05004	0.0e+00	5.644528	Interferon-related developmental regulator (IFRD)
PF00038	PF00038	0.0e+00	5.535107	Intermediate filament protein
PF00053	PF00053	0.0e+00	5.458336	Laminin EGF-like (Domains III and V)
PF04732	PF04732	1.0e-07	5.419825	Intermediate filament head (DNA binding) region
PF02893	PF02893	1.0e-07	5.336420	GRAM domain
PF00076	PF00076	1.0e-07	5.307513	RNA recognition motif. (a.k.a. RRM, RBD, or RNP domain)
PF08337	PF08337	1.0e-07	5.272545	Plexin cytoplasmic RasGAP domain
PF02096	PF02096	2.0e-07	5.183030	60Kd inner membrane protein
PF00152	PF00152	6.0e-07	4.989507	tRNA synthetases class II (D, K and N)
PF02210	PF02210	1.7e-06	4.783359	Laminin G domain
PF02779	PF02779	2.0e-06	4.748459	Transketolase, pyrimidine binding domain
PF00884	PF00884	3.4e-06	4.646329	Sulfatase
PF01485	PF01485	3.6e-06	4.631397	IBR domain

Downregulated Domains

```r
# Domain Enrichments
knitr::kable(Domains$DownRegulated_Domain)
```

	Domain	Pvalue	Zvalue	Info
PF03950	PF03950	0.0002850	-3.628604	tRNA synthetases class I (E and Q), anti-codon binding domain
PF08725	PF08725	0.0002953	-3.619413	Integrin beta cytoplasmic domain
PF07970	PF07970	0.0003411	-3.581933	Endoplasmic reticulum vesicle transporter
PF05821	PF05821	0.0004712	-3.496608	NADH-ubiquinone oxidoreductase ASHI subunit (CI-ASHI or NDUFB8)
PF04557	PF04557	0.0005325	-3.463845	Glutaminyl-tRNA synthetase, non-specific RNA binding region part 2
PF04558	PF04558	0.0005325	-3.463845	Glutaminyl-tRNA synthetase, non-specific RNA binding region part 1
PF00749	PF00749	0.0013734	-3.200191	tRNA synthetases class I (E and Q), catalytic domain
PF14913	PF14913	0.0026132	-3.009915	DPCD protein family
PF12755	PF12755	0.0026762	-3.002674	Vacuolar 14 Fab1-binding region
PF10498	PF10498	0.0026917	-3.000920	Intra-flagellar transport protein 57
PF10381	PF10381	0.0040001	-2.878150	Autophagocytosis associated protein C-terminal
PF04427	PF04427	0.0048895	-2.814226	Brix domain
PF00639	PF00639	0.0051955	-2.794654	PPIC-type PPIASE domain
PF03542	PF03542	0.0056809	-2.765655	Tuberin
PF14808	PF14808	0.0058558	-2.755749	TMEM164 family
PF11838	PF11838	0.0061584	-2.739227	ERAP1-like C-terminal domain
PF07965	PF07965	0.0064120	-2.725933	Integrin beta tail domain

	Domain	Pvalue	Zvalue	Info
PF14927	PF14927	0.0064729	-2.722814	Neurensin
PF11916	PF11916	0.0065669	-2.718044	Vacuolar protein 14 C-terminal Fig4p binding
PF01549	PF01549	0.0070537	-2.694297	ShK domain-like

Events are annotated with domains by using a sparse Matrix. This code shows how to access this annotation matrix.

```
data(DomainsEvents)
str(DomainsEvents)
```

```
## Formal class 'dgCMatrix' [package "Matrix"] with 6 slots
##   ..@ i       : int [1:130186] 3153 431 1091 1813 37 2885 4643 4674 877 1164 ...
##   ..@ p       : int [1:141773] 0 1 1 4 8 11 12 14 14 15 ...
##   ..@ Dim     : int [1:2] 5505 141772
##   ..@ Dimnames:List of 2
##   .. ..$ : chr [1:5505] "PF00001" "PF00002" "PF00003" "PF00004" ...
##   .. ..$ : chr [1:141772] "ENSG00000126215_9_PathA" "ENSG00000075711_32_PathA" "ENSG00000108509_3_Pa
##   ..@ x       : num [1:130186] 1 1 1 1 1 1 1 1 1 1 ...
##   ..@ factors : list()
```

We can get a subset of this matrix with the domains that are found to be upregulated or downregulated.

```
UpMatrix <- DomainsEvents[rownames(Domains$UpRegulated_Domain),]
UpMatrix <- UpMatrix[,which(colSums(UpMatrix) > 0)]

DnMatrix <- DomainsEvents[rownames(Domains$DownRegulated_Domain),]
DnMatrix <- DnMatrix[,which(colSums(DnMatrix) > 0)]
```

We can get the number of genes annotated to each of the domains

```
rowSums(UpMatrix)
```

```
## PF00052 PF08373 PF00400 PF06008 PF06009 PF02780 PF04836 PF05004 PF00038
##      50      32    1522      35      40      51      22      25     112
## PF00053 PF04732 PF02893 PF00076 PF08337 PF02096 PF00152 PF02210 PF02779
##     188      14     113     815      72      11      18     250      83
## PF00884 PF01485
##      74      82
```

```
rowSums(DnMatrix)
```

```
## PF03950 PF08725 PF07970 PF05821 PF04557 PF04558 PF00749 PF14913 PF12755
##      23      37      14       9      15      15      28       8      11
## PF10498 PF10381 PF04427 PF00639 PF03542 PF14808 PF11838 PF07965 PF14927
##       7       7      27       6      23       9      40      39       3
## PF11916 PF01549
##       6       8
```

Some of the domains are ubiquitous (i.e. many genes are annotated to them).

Events related with Upregulated Domains

It is interesting to find out which are the representative genes within each domain according to the p.value of their splicing events.

```
library(doBy)
namesEventsDomains <- unlist(strsplit(colnames(UpMatrix),"_Path"))[c(TRUE,FALSE)]
codeEvent <- match(namesEventsDomains, rownames(Events))

for (n in 1:nrow(UpMatrix)) {
  df  <- Events[sort(codeEvent[which(UpMatrix[n,]>0)])[1:15],]
  df  <- df[!is.na(df[,1]),]
  colnames(df)  <- c("Symbol","EnsemblID","Type","Number","Position","Z.value","P.value")
  Output <-  summaryBy(P.value ~ Symbol, FUN = min, data = df, id = ~ Position + Type)
  selected <- which(Output$P.value.min < 0.05)
  if(length(selected) >0) {
    Output <-  Output[selected,,drop = FALSE]
    cat(sprintf("\n#### %s ####\n",rownames(UpMatrix)[n]))
    print(knitr::kable(Output))
  } else {
    cat(sprintf("\n#### %s ####\n",rownames(UpMatrix)[n]))
    cat("\nNo events with significantly altered domains.\n")
  }
}
```

PF00052

Symbol	P.value.min	Position	Type
HSPG2	0.0071701	1:22174182-22174451	Retained Intron
LAMA5	0.0001224	20:60893899-60898912	Alternative First Exon

PF08373

	Symbol	P.value.min	Position	Type
1	FASTK	0.000000	7:150776587-150777877	Complex Event
3	TBRG4	0.022787	7:45142932-45143807	Alternative Last Exon

PF00400

Symbol	P.value.min	Position	Type
BCAS3	0	17:58964411-58967103	Alternative First Exon
DCAF6	0	1:167973771-168007726	Complex Event
NBEAL1	0	2:204055015-204082073	Alternative Last Exon
PLRG1	0	4:155458438-155459234	Alternative First Exon
RPTOR	0	17:78599491-78681799	Cassette Exon
SEC13	0	3:10342645-10345718	Complex Event
WDR77	0	1:111991352-111991874	Alternative First Exon

PF06008

Symbol	P.value.min	Position	Type
LAMA5	0.0001224	20:60893899-60898912	Alternative First Exon

PF06009

Symbol	P.value.min	Position	Type
LAMA5	0.0001224	20:60893899-60898912	Alternative First Exon
TPM3	0.0002883	1:154148604-154155479	Complex Event

PF02780

Symbol	P.value.min	Position	Type
PDHB	6e-07	3:58416570-58417355	Complex Event
TKT	0e+00	3:53258730-53259947	Complex Event

PF04836

	Symbol	P.value.min	Position	Type
2	IFRD2	6e-07	3:50326231-50326694	Alternative Last Exon

PF05004

	Symbol	P.value.min	Position	Type
2	IFRD2	6e-07	3:50326231-50326694	Alternative Last Exon

PF00038

	Symbol	P.value.min	Position	Type
1	KRT18	0.0055119	12:53342670-53343374	Alternative First Exon
3	KRT80	0.0000172	12:52579163-52579533	Alternative First Exon
4	LMNA	0.0004448	1:156084513-156084838	Complex Event
5	VIM	0.0000000	10:17272649-17275681	Alternative First Exon

PF00053

Symbol	P.value.min	Position	Type
AGRN	0.0054115	1:982707-983067	Alternative First Exon
HSPG2	0.0071701	1:22174182-22174451	Retained Intron
LAMA5	0.0001224	20:60893899-60898912	Alternative First Exon
LAMB2	0.0000317	3:49159596-49160485	Complex Event
MEGF11	0.0016379	15:66416237-66545995	Complex Event

PF04732

	Symbol	P.value.min	Position	Type
2	VIM	0	10:17272649-17275681	Alternative First Exon

PF02893

Symbol	P.value.min	Position	Type
GRAMD1A	0.0251189	19:35485688-35500233	Alternative First Exon
SBF1	0.0000614	22:50893910-50895102	Alternative First Exon
SBF2	0.0000363	11:9830459-9834181	Alternative First Exon
TBC1D8	0.0036033	2:101644807-101646238	Alternative Last Exon
TBC1D8B	0.0162141	X:106096751-106108857	Cassette Exon
TBC1D9B	0.0022355	5:179321459-179331812	Cassette Exon

PF00076

Symbol	P.value.min	Position	Type
EIF3B	0	7:2414163-2415162	Retained Intron
HNRNPA2B1	0	7:26232115-26235529	Alternative Last Exon
HNRNPDL	0	4:83347190-83348344	Cassette Exon
NONO	0	X:70503544-70510641	Complex Event
PSPC1	0	13:20251864-20304484	Complex Event
RBM42	0	19:36122233-36128543	Alternative Last Exon
RBM47	0	4:40440815-40468662	Cassette Exon
SRSF2	0	17:74730385-74731218	Complex Event
SRSF5	0	14:70235907-70237257	Complex Event
SRSF7	0	2:38977156-38978424	Alternative First Exon
TRA2A	0	7:23561326-23571595	Alternative First Exon

PF08337

	Symbol	P.value.min	Position	Type
1	PLXNB1	0.0352431	3:48454478-48455167	Complex Event
2	PLXNB2	0.0039110	22:50720436-50721309	Complex Event
4	PLXND1	0.0000000	3:129284835-129288800	Alternative Last Exon

PF02096

Symbol	P.value.min	Position	Type
COX18	4.30e-06	4:73933746-73935409	Complex Event
OXA1L	8.44e-05	14:23235928-23236618	Complex Event

PF00152

	Symbol	P.value.min	Position	Type
1	DARS	8.82e-05	2:136736844-136743062	Complex Event
3	NARS	0.00e+00	18:55269587-55270175	Retained Intron

PF02210

Symbol	P.value.min	Position	Type
CELSR1	0.0105847	22:46772958-46860242	Alternative First Exon
COL5A1	0.0022157	9:137717638-137722022	Complex Event
FAT1	0.0085019	4:187629517-187645009	Alternative First Exon
HSPG2	0.0071701	1:22174182-22174451	Retained Intron
LAMA5	0.0001224	20:60893899-60898912	Alternative First Exon
SLIT1	0.0011689	10:98758105-98819288	Complex Event

PF02779

	Symbol	P.value.min	Position	Type
2	PDHB	6e-07	3:58416570-58417355	Complex Event
3	TKT	0e+00	3:53258730-53259947	Complex Event

PF00884

Symbol	P.value.min	Position	Type
ARSA	0.0000962	22:51065984-51066583	Alternative First Exon
ARSD	0.0000608	X:2822959-2825673	Complex Event
ARSE	0.0004216	X:2878419-2882305	Alternative 3' Splice Site
SGSH	0.0000000	17:78188522-78190991	Cassette Exon
SULF2	0.0000008	20:46331263-46386075	Complex Event

PF01485

	Symbol	P.value.min	Position	Type
1	ANKIB1	0.0000000	7:92020480-92025790	Complex Event
2	ARIH2	0.0000006	3:49008100-49011249	Complex Event
3	CUL9	0.0000038	6:43183896-43188219	Alternative 5' Splice Site
7	RNF31	0.0232970	14:24627376-24629616	Cassette Exon

Events related with Downregulated Domains

Similarly, for the downregulated domains,

```
library(doBy)
namesEventsDomains <- unlist(strsplit(colnames(DnMatrix),"_Path"))[c(TRUE,FALSE)]
codeEvent <- match(namesEventsDomains, rownames(Events))

for (n in 1:nrow(DnMatrix)) {
```

```
  df  <- Events[sort(codeEvent[which(DnMatrix[n,]>0)])[1:15],]
  df  <- df[!is.na(df[,1]),]
  colnames(df)  <- c("Symbol","EnsemblID","Type","Number","Position","Z.value","P.value")
  Output <-  summaryBy(P.value ~ Symbol, FUN = min, data = df, id = ~ Position + Type)
  selected <- which(Output$P.value.min < 0.05)
  if(length(selected) >0) {
    Output <-  Output[selected,,drop = FALSE]
    cat(sprintf("\n#### %s ####\n",rownames(DnMatrix)[n]))
    print(knitr::kable(Output))
  } else {
    cat(sprintf("\n#### %s ####\n",rownames(DnMatrix)[n]))
    cat("\nNo events with significantly altered domains.\n")
  }
}
```

PF03950

Symbol	P.value.min	Position	Type
QARS	0.0417099	3:49135786-49136125	Complex Event

PF08725

Symbol	P.value.min	Position	Type
ITGB1	0	10:33224420-33247124	Alternative First Exon

PF07970

Symbol	P.value.min	Position	Type
ERGIC1	0.0007627	5:172341717-172351064	Complex Event
ERGIC3	0.0000000	20:34130072-34130349	Alternative First Exon

PF05821

Symbol	P.value.min	Position	Type
NDUFB8	3.05e-05	10:102286156-102289614	Alternative Last Exon

PF04557

Symbol	P.value.min	Position	Type
QARS	0.0417099	3:49135786-49136125	Complex Event

PF04558

Symbol	P.value.min	Position	Type
QARS	0.0417099	3:49135786-49136125	Complex Event

Symbol	P.value.min	Position	Type

PF00749

	Symbol	P.value.min	Position	Type
2	QARS	0.0417099	3:49135786-49136125	Complex Event

PF14913

Symbol	P.value.min	Position	Type
DPCD	0.0074906	10:103348089-103354458	Alternative Last Exon

PF12755

Symbol	P.value.min	Position	Type
KIAA0368	8.2e-06	9:114213705-114246337	Complex Event

PF10498

Symbol	P.value.min	Position	Type
IFT57	0.0159676	3:107937382-107941207	Complex Event

PF10381

Symbol	P.value.min	Position	Type
ATG3	0.0016924	3:112251369-112253184	Retained Intron

PF04427

	Symbol	P.value.min	Position	Type
2	PPAN	0.0217555	19:10221621-10225414	Complex Event

PF00639

Symbol	P.value.min	Position	Type
PIN1	0.0141749	19:9958706-9959871	Alternative Last Exon

PF03542

Symbol	P.value.min	Position	Type
TSC2	1e-07	16:2108748-2112518	Complex Event

PF14808

Symbol	P.value.min	Position	Type
TMEM164	0.0001721	X:109245915-109247392	Complex Event

PF11838

No events with significantly altered domains.

PF07965

Symbol	P.value.min	Position	Type
ITGB1	0	10:33224420-33247124	Alternative First Exon

PF14927

No events with significantly altered domains.

PF11916

No events with significantly altered domains.

PF01549

No events with significantly altered domains.

GO enrichment analysis

It can be interesting to see the functions of the genes that present differential alternative splicing. The GO enrichment analysis requires an additional step if compared with the analysis of gene expression. Since a gene may have several splicing events, the events must be summarized for every gene to have a single value in order to perform hte analysis. One reasonable possibility is to take minimum p.value of all the events within a gene.

```
Genes <- Events
colnames(Genes)  <- c("Symbol","EnsemblID","Type","Number","Position","Z.value","P.value")
GenesShort  <- summaryBy(P.value ~ EnsemblID, FUN = min,
                         data = Genes, id = ~ Position + Type)
names <- GenesShort$EnsemblID
GenesShort <- GenesShort$P.value.min
names(GenesShort) <- names
```

Now we can run a GO enrichment analysis

```
# GO enrichment analysis
library(org.Hs.eg.db)
library(topGO)

# Select anyone with AS (p.value < 1e-3)
GOdata <- new("topGOdata", ontology = "BP", allGenes = GenesShort,
```

```
              geneSel = function(x) x  < 1e-3,
              description = "Test", annot = annFUN.org, mapping = "org.Hs.eg.db",
              ID = "Ensembl")
resultFisher <- runTest(GOdata, algorithm = "weight01", statistic = "fisher")
```

And display the results.

```
knitr::kable(GenTable(GOdata, wFisher = resultFisher, topNodes = 20))
```

GO.ID	Term	Annotated	Significant	Expected	wFisher
GO:0043488	regulation of mRNA stability	94	35	14.74	4.6e-06
GO:0043687	post-translational protein modification	308	76	48.30	0.00011
GO:0090263	positive regulation of canonical Wnt sig...	85	26	13.33	0.00040
GO:0090557	establishment of endothelial intestinal ...	6	5	0.94	0.00049
GO:0051437	positive regulation of ubiquitin-protein...	53	18	8.31	0.00078
GO:0016925	protein sumoylation	86	26	13.49	0.00080
GO:0051262	protein tetramerization	66	18	10.35	0.00091
GO:0007050	cell cycle arrest	159	41	24.93	0.00094
GO:0043928	exonucleolytic nuclear-transcribed mRNA ...	19	9	2.98	0.00116
GO:0032508	DNA duplex unwinding	40	14	6.27	0.00119
GO:0000398	mRNA splicing, via spliceosome	206	56	32.30	0.00144
GO:0048208	COPII vesicle coating	43	15	6.74	0.00154
GO:0048025	negative regulation of mRNA splicing, vi...	17	8	2.67	0.00231
GO:0006554	lysine catabolic process	7	5	1.10	0.00263
GO:0034427	nuclear-transcribed mRNA catabolic proce...	5	4	0.78	0.00264
GO:0010975	regulation of neuron projection developm...	237	40	37.17	0.00264
GO:0006369	termination of RNA polymerase II transcr...	37	13	5.80	0.00290
GO:0051974	negative regulation of telomerase activi...	11	6	1.72	0.00333
GO:0008152	metabolic process	6401	1075	1003.79	0.00348
GO:0018279	protein N-linked glycosylation via aspar...	163	39	25.56	0.00367

These functions are in line with the known functions of SRSF1 (for a thorough review, see *italics* The translational landscape of the splicing factor SRSF1 and its role in mitosis *italics*, Caceres et. al., eLife, DOI: 10.7554/eLife.02028.001).

Dependencies

- aroma.affymetrix Microarray pre-processing
- limma Statistical framework
- Matrix Sparse Matrices
- matrixStats Statistical functions on matrices
- dcGOR Protein Domains

Session Info

```
sessionInfo()
```

```
## R version 3.3.0 beta (2016-04-04 r70420)
```

```
## Platform: x86_64-apple-darwin13.4.0 (64-bit)
## Running under: OS X 10.11.4 (El Capitan)
##
## locale:
## [1] en_US.UTF-8/en_US.UTF-8/en_US.UTF-8/C/en_US.UTF-8/en_US.UTF-8
##
## attached base packages:
## [1] parallel  stats4    stats     graphics  grDevices utils     datasets
## [8] methods   base
##
## other attached packages:
##  [1] topGO_2.23.4          SparseM_1.7           GO.db_3.3.0
##  [4] graph_1.49.1          org.Hs.eg.db_3.3.0    AnnotationDbi_1.33.13
##  [7] IRanges_2.5.46        S4Vectors_0.9.52      Biobase_2.31.3
## [10] BiocGenerics_0.17.5   doBy_4.5-15           EventPointer_1.0
## [13] dcGOR_1.0.6           dnet_1.0.8            supraHex_1.9.0
## [16] hexbin_1.27.1         igraph_1.0.1          matrixStats_0.50.2
## [19] Matrix_1.2-5          limma_3.27.19         biomaRt_2.27.2
## [22] aroma.light_3.1.1     aroma.affymetrix_3.0.0 aroma.core_3.0.0
## [25] R.devices_2.14.0      R.filesets_2.10.0     R.utils_2.3.0
## [28] R.oo_1.20.0           ggplot2_2.1.0         affxparser_1.43.2
## [31] R.methodsS3_1.7.1     knitr_1.12.3          BiocInstaller_1.21.4
##
## loaded via a namespace (and not attached):
##  [1] Rcpp_0.12.4.5    ape_3.4          lattice_0.20-33  listenv_0.6.0
##  [5] digest_0.6.9     plyr_1.8.3       RSQLite_1.0.0    evaluate_0.8.3
##  [9] R.huge_0.9.0     highr_0.5.1      Rgraphviz_2.15.0 rmarkdown_0.9.5
## [13] labeling_0.3     stringr_1.0.0    RCurl_1.95-4.8   munsell_0.4.3
## [17] base64enc_0.1-4  aroma.apd_0.6.0  R.rsp_0.21.0     globals_0.6.1
## [21] htmltools_0.3.5  DNAcopy_1.45.0   codetools_0.2-14 XML_3.98-1.4
## [25] future_0.13.0    MASS_7.3-45      bitops_1.0-6     grid_3.3.0
## [29] nlme_3.1-127     gtable_0.2.0     DBI_0.3.1        magrittr_1.5
## [33] formatR_1.3      scales_0.4.0     stringi_1.0-1    PSCBS_0.61.0
## [37] tools_3.3.0      R.cache_0.12.0   yaml_2.1.13      colorspace_1.2-6
```

Authors

Juan Pablo Romero (jpromero@ceit.es) : Bioinformatics Group, CEIT, San Sebastian, Spain Angel Rubio : Bioinformatics Group, CEIT, San Sebastian, Spain Ander Muniategui: Bioinformatics Group, CEIT, San Sebastian, Spain
