# Supplementary figures and images for: EventPointer: an effective identification of alternative splicing events using junction arrays
Source: BMC Genomics. 2016 Jun 17;17:467. doi: 10.1186/s12864-016-2816-x (PMC4912780; doi:10.1186/s12864-016-2816-x)

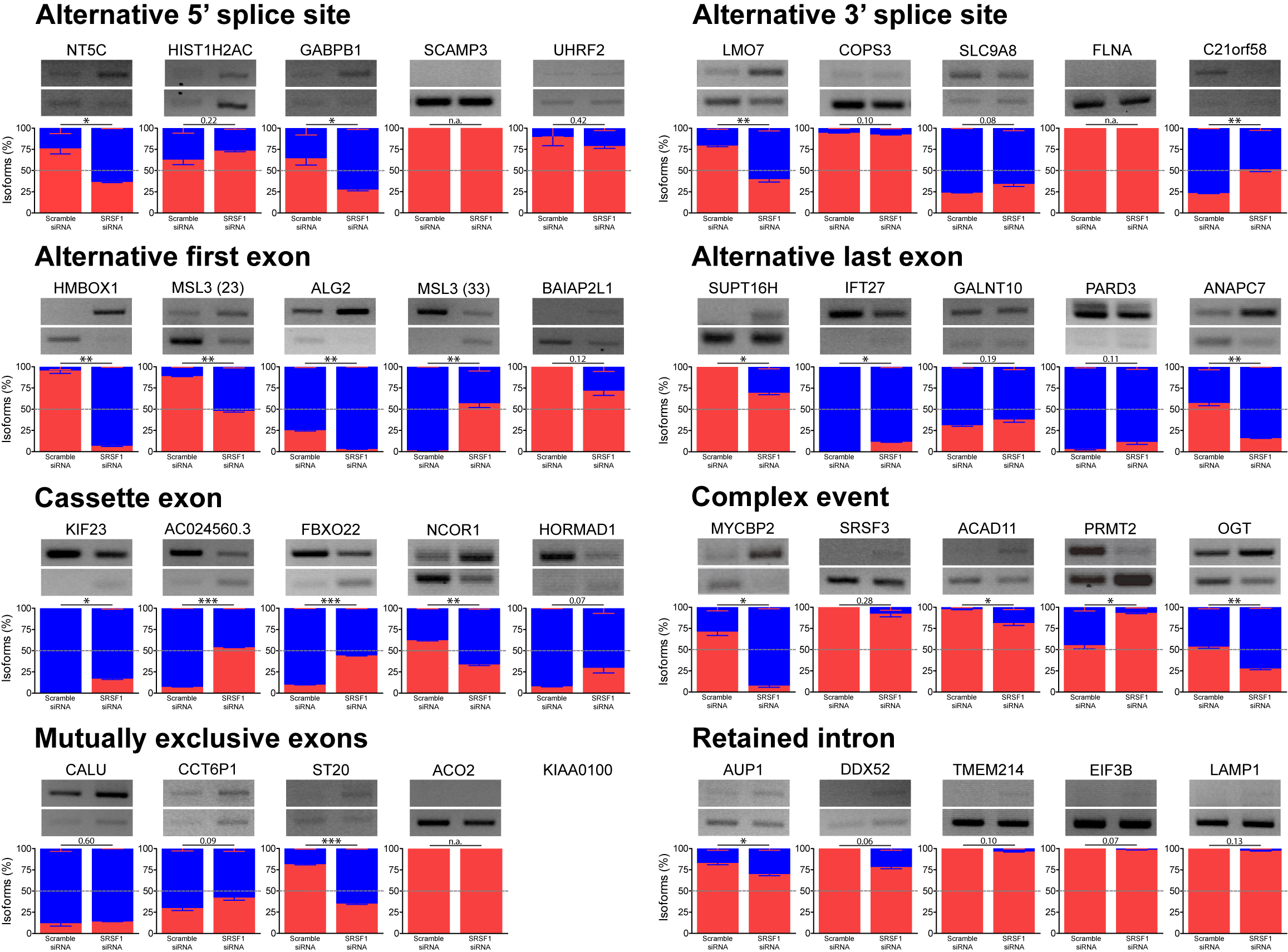

Supplement: Additional file 3: — PCR images and relative concentrations of the isoforms based on the PCR image. The red bar corresponds to the shorter isoform and the blue bar to the longer isoform. For the event in KIAA0100, it was not possible to get PCR results, and thus the image is not included. (TIF 1465 kb) [file 12864_2016_2816_MOESM3_ESM.tif]

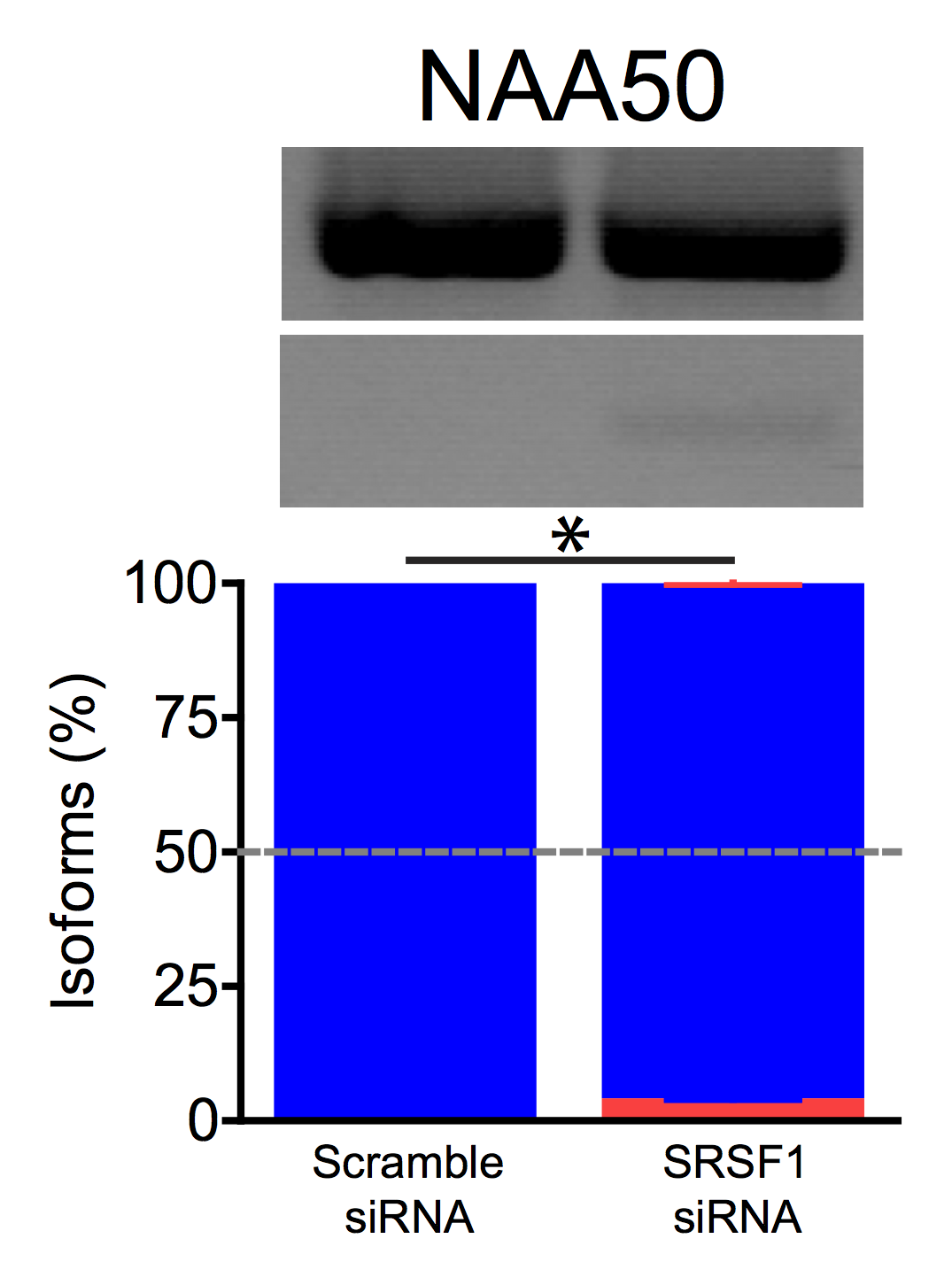

Supplement: Additional file 6: — PCR images and relative concentrations of the NAA50 gene detected by TAC 3.0. The red bar corresponds to the shorter isoform and the blue bar to the longer isoform. (PNG 166 kb) [file 12864_2016_2816_MOESM6_ESM.png]

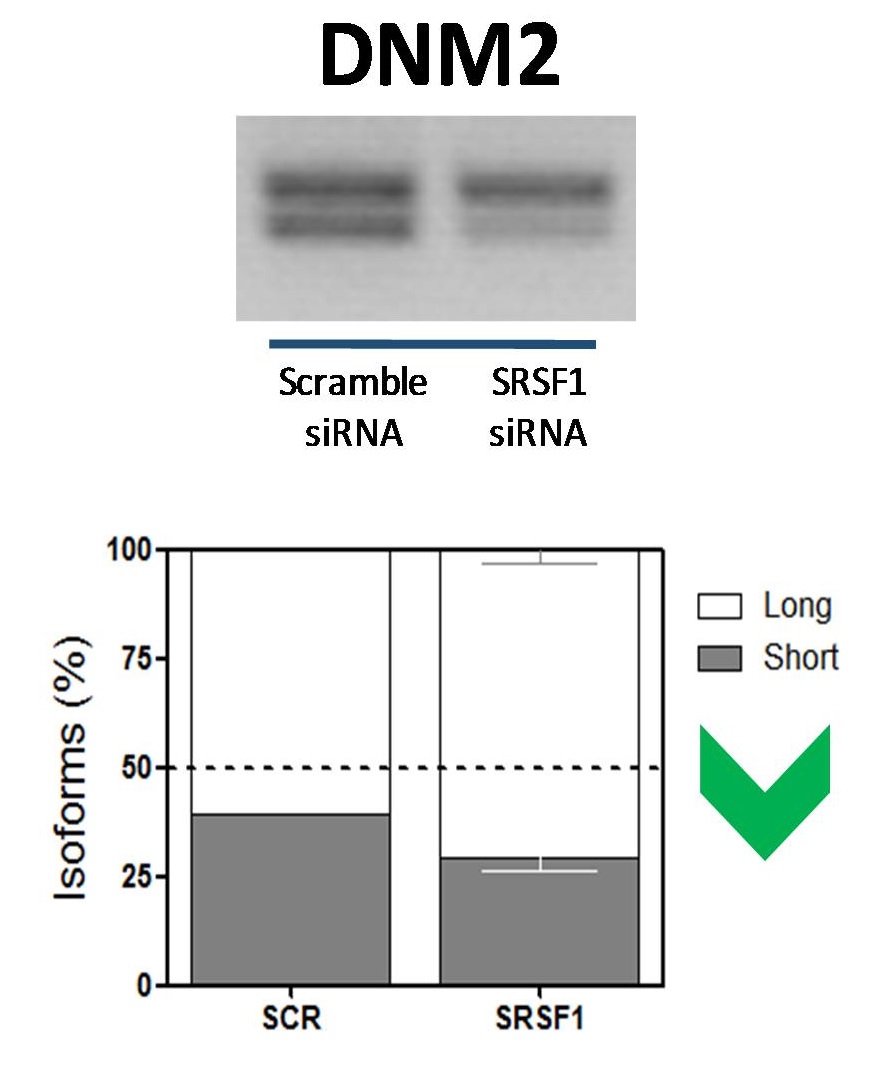

Supplement: Additional file 7: — PCR images and relative concentrations of the isoforms based on the PCR image of gene DNM2. The grey bar corresponds to the shorter isoform and the white bar to the longer isoform. (JPG 92 kb) [file 12864_2016_2816_MOESM7_ESM.jpg]
