# Supplementary material for: EventPointer: an effective identification of alternative splicing events using junction arrays
Source: BMC Genomics. 2016 Jun 17;17:467. doi: 10.1186/s12864-016-2816-x (PMC4912780; doi:10.1186/s12864-016-2816-x)
Supplement: Additional file 4: — Table with primers used for validation and sizes of the expected splicing variants. (DOCX 24 kb) [file 12864_2016_2816_MOESM4_ESM.docx]

Table S1. Primers used for validation and sizes of the sizes of the expected splicing variants

| **Gene** | **Direction** | **Sequence (5'→3')** | **Product sizes (bp)** |
| --- | --- | --- | --- |
| **MYCBP2** | **Forward** | **CAGTGCCCAAGGATTTGATT** | **119 / 299** |
|  | **Reverse** | **TGCTGTGTTTAGAGGACAGCTC** |  |
| **KIF23** | **Forward** | **TGTGGCTAATCCCTTGGTCA** | **98 / 235** |
|  | **Reverse** | **GGCATCAACCTCACACTGTA** |  |
| **AC024560.3** | **Forward** | **CCTACCAGGTCACACACTGT** | **98 / 242** |
|  | **Reverse** | **CCACCAAATGCACGCTGATA** |  |
| **FBXO22** | **Forward** | **GTTGAGTAACCTGGCGGAG** | **101 / 240** |
|  | **Reverse** | **ACTGTATGTGGTAAGATGCGAAC** |  |
| **SRSF3** | **Forward** | **GAGTCCTCCACCTCGTCG** | **125 / 581** |
|  | **Reverse** | **GGGACGGCTTGTGATTTCTC** |  |
| **SUPT16H** | **Forward** | **CTCGCGTGATTCTCGGAAC** | **166 / 193** |
|  | **Reverse1** | **TCAACACCCACTGATACAACA** |  |
|  | **Reverse2** | **CTGTTAAGGGGCAGCGTTC** |  |
| **HMBOX1** | **Forward1** | **CCCAGATGAAGCAAAGAGGG** | **152 / 200** |
|  | **Forward2** | **ACTTGAACTCTCCCTGGAACA** |  |
|  | **Reverse** | **TGGCTCTCCTCTTGATCTCC** |  |
| **ACAD11** | **Forward** | **AAAATGGTGGAGCAGTGGAG** | **111 / 286** |
|  | **Reverse** | **ACAAGAATCATGCTGTGCTGT** |  |
| **NCOR1** | **Forward** | **GGTCTTGGCCCAAAAGTTGA** | **177 / 204** |
|  | **Reverse** | **GAGCTGATCATAACGCTGGC** |  |
| **AUP1** | **Forward** | **GGAACTCAGTCCCTACCCAC** | **150 / 212** |
|  | **Reverse** | **TGCGTATTCATATAGTGCTTGCT** |  |
| **IFT27** | **Forward1** | **AAGAACAATCCGCGCTGATC** | **173 / 211** |
|  | **Forward2** | **GAGCAGTGGACTCAGCTGA** |  |
|  | **Reverse** | **TCTGTCTTCTCCGGTTGTGC** |  |
| **GALNT10** | **Forward** | **GATCCCTCCAGAACTGCAGA** | **152 / 227** |
|  | **Reverse1** | **TCCTGTAGATATGGCCCACC** |  |
|  | **Reverse2** | **TTTCATACTGCTCCCCTCCC** |  |
| **PARD3** | **Forward** | **CCCATTGAAACAGCGTTGGA** | **179 / 237** |
|  | **Reverse1** | **CAGGACGCAGGTTACAGGAA** |  |
|  | **Reverse2** | **ATCATGGGAGCTGGAAGAGG** |  |
| **PRMT2** | **Forward** | **CTCAGGCTCCTGGAAAGGAC** | **244 / 353** |
|  | **Reverse** | **CCACAAACTCCTCTGGCTGT** |  |
| **HORMAD1** | **Forward** | **CCTGTATCACGTATTTGAGGGG** | **151 / 215** |
|  | **Reverse** | **TGTCTGAGGATCTTCTGGGT** |  |
| **ANAPC7** | **Forward** | **GCTGTCAATGAGTATCAGGAGG** | **100 / 164** |
|  | **Reverse1** | **TCTCCATCTTCTGCATCCCC** |  |
|  | **Reverse2** | **CTGCTTTCAACCATGGACACA** |  |
| **OGT** | **Forward1** | **CTGTGTTCGCAGTGACCT** | **156 / 248** |
|  | **Forward2** | **ATGCTGCAGGGTCACTTTTG** |  |
|  | **Reverse** | **AGCCAAATTTCCCCTTGTGC** |  |
| **MSL3 (23)** | **Forward** | **CCGGCAGCAGGACACATC** | **150 / 193** |
|  | **Reverse1** | **GTTCTTCTCCCTTCAAAGCCA** |  |
|  | **Reverse2** | **GCATGACAGCACTTTGGACA** |  |
| **NT5C** | **Forward** | **TGCTGAAGTACCACCACTGT** | **103 / 183** |
|  | **Reverse** | **ACCGTCTTGTCCCTTGTCAG** |  |
| **ALG2** | **Forward** | **AGGAGTTCGACGTGGTAGTG** | **81 / 240** |
|  | **Reverse** | **GGATCTTCTTCCGCCGTCTA** |  |
| **MSL3 (33)** | **Forward1** | **CCAGAAGCATTGCAGTCTCT** | **151 / 235** |
|  | **Forward2** | **AACAGTCTTGAGGCAGTCTTT** |  |
|  | **Reverse** | **TCCCTTCAAAGCCAGCAAAC** |  |
| **BAIAP2L1** | **Forward1** | **CGTCCTCCTCCCAACTTCTT** | **193 / 260** |
|  | **Forward2** | **TTTGACATTTTCGCAGCGGA** |  |
|  | **Reverse** | **AATTTCGCAGCCCAGGATTG** |  |
| **HIST1H2AC** | **Forward** | **GGCGTCCTTCCTAACATCCA** | **130 / 246** |
|  | **Reverse** | **GGCACTTAGGATCCTGGCAT** |  |
| **DDX52** | **Forward** | **GGTGTGAACTTGGTGATCAACT** | **93 / 271** |
|  | **Reverse** | **TCCCTTATTCCCTGCTCTTCC** |  |
| **TMEM214** | **Forward** | **GGGAGCTACGTGGGATCATC** | **176 / 417** |
|  | **Reverse** | **GGCAATCTTGGGCTTGTCTT** |  |
| **GABPB1** | **Forward** | **ACACAAACCCAGAGAGTCCT** | **115 / 151** |
|  | **Reverse** | **TGAACAGCAGATACACCCGT** |  |
| **EIF3B** | **Forward1** | **AGATGTTCGACAAGCAGCAG** | **154 / 208** |
|  | **Forward2** | **TGTTGCTGTGCTTGTTTCCA** |  |
|  | **Reverse** | **GAAGCCATGTAGTGCTCTGC** |  |
| **LAMP1** | **Forward1** | **CGGTGACAAGGCTTCTCAAC** | **189 / 244** |
|  | **Forward2** | **ACACAACGCCTTTTATAGACAAG** |  |
|  | **Reverse** | **TGGCGTCAGGAAGAATTGTAT** |  |
| **LMO7** | **Forward** | **AAAAGAGTGGGAGGAAGCCA** | **171 / 252** |
|  | **Reverse** | **TCGGAGAAATCAGTTGTTACAGA** |  |
| **SCAMP3** | **Forward** | **CCACCATGTACTACCTCTGGA** | **160 / 195** |
|  | **Reverse** | **TACCAGCAGACAAAGGAGCA** |  |
| **UHRF2** | **Forward** | **GCCCTAAACTGTGATGCTCC** | **159 / 250** |
|  | **Reverse** | **TCCAAGGAGCAGGTTCAACA** |  |
| **COPS3** | **Forward** | **CCATCAGCTAACAAATGCACTTG** | **117 / 185** |
|  | **Reverse** | **GCATGTATTGAGGTCAGCTGG** |  |
| **SLSC9A8** | **Forward** | **CCATCACCCTGTTTGCTGTT** | **176 / 211 / 259** |
|  | **Reverse** | **AAGACCAGCATGTTGAGCAC** |  |
| **FLNA** | **Forward** | **CCGACATCCCCATCAACATC** | **235 / 252** |
|  | **Reverse** | **GCATCCCCAATTTCCGACTG** |  |
| **C21orf58** | **Forward** | **GCGAGTAACAGAACCAGGGA** | **160 / 240** |
|  | **Reverse** | **CGTTCTGCCGTTCTTGCTC** |  |
| **CALU** | **Forward** | **CCTCAGCTCAGTGACAAGGT** | **166 / 243** |
|  | **Reverse1** | **CTGTCGCTCTACATCCTCGT** |  |
|  | **Reverse2** | **ACCCATCTTTATCCGCGTCT** |  |
| **CCT6P1** | **Forward** | **CTGTGTGGTTCCGGTGCT** | **171 / 275** |
|  | **Reverse** | **AGCTGTTTCTTTACACAATCGTT** |  |
| **ST20** | **Forward** | **CTCTTTCCTCCGGCTCTTCC** | **159 / 250** |
|  | **Reverse** | **GGAGACAGAGGTTGCAGTGA** |  |
| **ACO2** | **Forward** | **CCTACTGGTGACTCGGCTG** | **243 / 432** |
|  | **Reverse** | **TTGCCTCGCTCAATTTCCTG** |  |
