# Supplementary material for: EventPointer: an effective identification of alternative splicing events using junction arrays
Source: BMC Genomics. 2016 Jun 17;17:467. doi: 10.1186/s12864-016-2816-x (PMC4912780; doi:10.1186/s12864-016-2816-x)
Supplement: Additional file 5: — Results for top-ranked events according to TAC 3.0. (DOCX 17 kb) [file 12864_2016_2816_MOESM5_ESM.docx]

Table S2. First events ranked according to the Splicing Index by TAC 3.0.

| Gene | Genomic Position | Splicing Index | Type | PSR/Junction |
| --- | --- | --- | --- | --- |
| HNRNPH3 | 13:70090931- 70102953 | 37.75 |  | Junction |
| NRG1 | 15:31496902- 32622558 | -24.66 |  | Junction |
| KIF23 | 3:69706585 - 69740764 | 16.67 |  | Junction |
| NRG1 | 15:31496902 - 32622558 | -16.49 | Cassette Exon | PSR |
| NOL8 | 6:95059640 - 95087876 | 15.97 |  | Junction |
| NAA50 | 14:113435307- 113465147 | 14.35 |  | Junction |
| DDAH1 | 8:85784168 - 86044046 | 13.45 | Cassette Exon | PSR |
| HNRNPH1 | 3:179041179- 179061785 | 13.03 |  | Junction |
| GREB1 | 17:11674242 - 11782914 | 13.02 |  | Junction |
| CYP2C9 | 2:33038100 - 33138722 | 12.39 |  | Junction |

Table S3. First events ranked according to the Splicing Index by AltAnalyze.

| Gene | Probeset Locations | ASPIRE |
| --- | --- | --- |
| NRG1 | chr8:32579560-32585467(+)\|chr8:32463201-32585467(+) | 0,723279976 |
| TXNRD1 | chr12:104703629-104703877(+)\|chr12:104684882-104705068(+) | -0,68285044 |
| NRG1 | chr8:32579363-32579560(+)\|chr8:32463201-32585467(+) | 0,651647335 |
| RP11-773D16.1 | chr10:81565894-81574353(-)\|chr10:81568735-81574353(-) | 0,639581295 |
| KIF23 | chr15:69733418-69737129(+)\|chr15:69733418-69738348(+) | 0,613240535 |
| HMBOX1 | chr8:28903817-28903933(+)\|chr8:28902960-28904875(+) | -0,606337523 |
| PARPBP | chr12:102548605-102549062(+)\|chr12:102547754-102558216(+) | -0,584156296 |
| FAM114A1 | chr4:38870167-38879692(+)\|chr4:38869455-38879692(+) | -0,580500032 |
| AC024560.3 | chr3:197348755-197350075(-)\|chr3:197348755-197349042(-) | -0,570439834 |
| MFF | chr2:228212100-228220393(+)\|chr2:228205096-228217230(+) | 0,561452427 |
