# Supplementary material for: EventPointer: an effective identification of alternative splicing events using junction arrays
Source: BMC Genomics. 2016 Jun 17;17:467. doi: 10.1186/s12864-016-2816-x (PMC4912780; doi:10.1186/s12864-016-2816-x)
Supplement: Additional file 8: — Supplementary methods. Detection and classification of events. (DOCX 63 kb) [file 12864_2016_2816_MOESM8_ESM.docx]

**Detection and Classification of events**

**Mapping.** The probes included in HTA 2.0 array from Affymetrix are mapped against the human transcriptome (Ensembl 75) using Bowtie 2.0. Multimapping probes, those that map against more than 3 genes, are removed since they are considered to be non-informative.

**Construction of the splicing graph.** The Splicing Graph (SG)[25] is a directed graph used to represent the structure of a gene. The nodes are here called pseudoexons. We define pseudoexon as a contiguous region of the genome that belongs to exactly the same set of transcripts for a given transcriptome (in our case Ensembl 74). For example, if an AS event is an alternative 5’ site, the exon involved is split into two contiguous, non-overlapping smaller pseudoexons.

In the SG, two nodes (pseudo-exons) are connected by an edge if there is at least one isoform that includes both nodes and these pseudoexons are contiguous in the mRNA of this isoform. In addition to the standard nodes, two additional nodes (start and end) are added to the graph. All the nodes (pseudoexons) that appear at the 5’ (3’) locus of any isoform are connected to the start (end) node.

In order to relate the mapped probes to the SG, all the nodes of the SG have been duplicated: one of the nodes (a-node) includes all the incoming edges and the other one (b-node) includes the outgoing ones. Both nodes are in turn, connected by an edge. This new directed graph is also bipartite: a-nodes are linked exclusively with b-nodes and b-nodes linked exclusively with a-nodes. Figure illustrates the construction of the SG. This duplication of the nodes in the SG allows setting a relationship between probes and edges: all the mapped probes in the array can be assigned to an edge in the SG of the genes. If the probes are mapped to a pseudo-exon, their corresponding edge links an a-node with a b-node. Junction probes (and probes mapped to the loci between two contiguous pseudo-exons in the genome) have links that bind b-nodes with a-nodes.

**Pruning and recovering of the splice graph.** The SGs (one per gene) are very complex and include many edges not supported by any probe on the array. This complexity makes it difficult to properly classify the events (in fact, most of them are unclassifiable). Additionally, it is nonsense to investigate an event if it has no probes mapped to it. It is desirable to simplify the SG by keeping only the edges that have probes mapped to them. In order to prune the graph on the SG, we introduce the concept of coherence: a SG is coherent if there is a path that connects the start node with every node and every node has a path that connects it with the end node.

The original SG is simplified by removing the edges that have no probes mapped to them unless this removal implies the loss of coherence in the graph. To illustrate this procedure, let us assume that there are no probes mapped to exon 4 and junctions E2-E4 and E4-E5 in the isoforms shown in Figure 6a of the main manuscript. Thus, the corresponding edges are removed from the SG (Figure 6b of the main manuscript). Let us assume, in addition, that there are no probes mapped to the E7-E8 junction (for example, because the included probes were mapped ubiquitously in the transcriptome). This edge is initially removed but is recovered in order to keep the coherence of the SG (Figure 6c of the main manuscript).

**Finding the splicing events**. A splicing event is defined (in this work) as a triplet of sets of edges {PR, P1, P2} that share the following characteristics: for any arbitrary network flow compatible with the graph, 1) the flow traversing the edges within a member of the triplet is identical and probably different to the flows in other members of the triplet, and 2) the flow traversing PR is the sum of the flows traversing P1 and P2. In order to avoid the ambiguity between P1 and P2, P1 is assigned to be the set of edges with the largest genomic length in the transcriptome (i.e. in a cassette event, P1 is the path that includes the skipping exon).

In order to clarify this definition, we will show some examples. In a cassette event, the subset PR includes at least the exons flanking the skipped exon, “P1” includes the skipped exon and the flanking junctions and “P2” includes exclusively the junction that skips the exon. Figure 6 shows a cassette of E7. For this event, PR is composed of E1, E6 and E8. P1 is composed of E7. Finally, P2 consists only of edge E6-E8.

It can be noted that, in the previous example, PR incudes not only exons E6 and E8 but also E1. The three exons E1, E6 and E8 are shared by all the isoforms and therefore, the flow that traverses them is identical and the three of them can be used as references.

Using the definition, it is possible to get all the events in a SG. In a graph, the flow incoming to a node must be identical to the flow outgoing from it (except for the start and end nodes). With the help of the incidence matrix of the SG, this arbitrary flow distribution can be obtained by solving:

(1)

$$\sum_{j|a_{ij}=1} v_{j}=\frac{\sum_{j|a_{ij}=-1} r_{ij}\cdot v_{j}}{\sum_{j|a_{ij}=\{-1,1\}} r_{ij}} \forall i$$

(2)

(3)

***A*** = {*a_ij_* = 1, -1, 0} is the incidence matrix of the directed graph SG *n* × *e*, where *n* and *e* are the number of nodes and edges, respectively. ***A****(*(n-2)* × *e)* is the incidence matrix excluding the start and end nodes. The entries *a_ij_* of the incidence matrix are 1, if edge *j* is incoming to node *i*, -1 if edge *j* is outgoing to node *i,* and 0 otherwise. The column vector **v** of size *e* x *1*represents the flow traversing each edge. The notation (·) is a logical bracket whose value is 1 if the expression inside the brackets is true and 0 otherwise. *r_ij_* are random numbers that follow any arbitrary distribution. Equation (1) imposes the continuity of the flows (i.e. the flow incoming to a node is identical to the flow exiting from it except for the start (source) and end (sink) nodes. Equation (2) imposes for every bifurcation a random assignment of the incoming flows to the outgoing flows. Finally, Equation (3) imposes that all the edges that exit the source node have flow equal to one. Taken together, equations (1) to (3) represent a linear system of equations. In fact, this is an algorithm to generate a random **v** that belongs to the null space of the incidence matrix.

Once the flows have been calculated, the triplets are determined as follows. If the flow is identical for several edges, all of them are part of the same set. After the edges are grouped into sets, it is possible to find triplets of sets in which the sum of the flow in two of the triplets (P1 and P2) equals the third one (PR). The same set can participate in different events as playing the PR role, but only at most on one as P1 or P2.

**Labeling the type of splicing event**. The splicing events can be classified into 7 major categories: cassette exons, alternative 3´donor site, alternative 5´donor site, intron retention, alternative last exon, alternative first exon and mutually exclusive exons. Any event not classified into them is considered to be a complex event. It is possible to label an event according to the structure of the subgraph of the SG that includes PR, P1 and P2.

Four different graph patterns are needed to classify the events. Figure 6 of the main manuscript shows that cassette exons, alternative 3' donor site, alternative 5' donor site and intron retentions share the same pattern. The lengths of junctions 1.b - 2.a and 2.b - 3.a are used to differentiate them. For cassette exons, both lengths must be greater than 1; in the case of retained introns, must be equal to 1; and for alternative 3' (5') donor sites junction 1.b - 2.a (2.b - 3.a) must be equal to one.

The rest of the events (alternative first exon, alternative last exon and mutually exclusive exons) can be identified by looking for the specific patterns shown in Figure 6 of the main manuscript.

**Summarizing the events.** Each of the events is composed of a triplet of probesets: the probes mapped to path 1, path 2 and the reference. For a specific event, there are isoforms that are not mapped to any of the paths in the event. However, if an isoform hits the event then, by construction, the isoform can be mapped to PR and *only to either* P1 or P2.

Within the analysis, we consider that the signal of a probe on an Affymetrix array is the product of the affinity of the probe and the sum of the concentrations of the isoforms interrogated by the probe. Therefore, if $y_{ij}$ is the logarithm of signal of the probe i in condition j then,

$y_{ij}=\log\left(Affy_{i}\tau_{j}\right)+\varepsilon_{ij}=\log\left(Affy_{1}\right)+\log\left(\tau_{j}\right)+\varepsilon_{ij}$ (4)

where$Affy_{i}$is the affinity of the probe, $\tau_{j}$is the sum of the concentrations of the transcripts interrogated by probe *i*, and$\varepsilon_{ij}$is an error term.

In EventPointer (as in most other methods that use Affymetrix technology), the values of the probe signals within a probeset are summarized to a single value using the median polish algorithm [26]. We have assumed that the model for a single probe is also valid for the summarized value of the probeset (i.e. the signal in the probeset is proportional to the concentration of the isoforms interrogated by the probeset).
